# An ortholog of *Plasmodium falciparum* chloroquine resistance transporter (PfCRT) plays a key role in maintaining the integrity of the endolysosomal system in *Toxoplasma gondii* to facilitate host invasion

**DOI:** 10.1371/journal.ppat.1007775

**Published:** 2019-06-06

**Authors:** L. Brock Thornton, Paige Teehan, Katherine Floyd, Christian Cochrane, Amy Bergmann, Bryce Riegel, Andrew J. Stasic, Manlio Di Cristina, Silvia N. J. Moreno, Paul D. Roepe, Zhicheng Dou

**Affiliations:** 1 Department of Biological Sciences, Clemson University, Clemson, South Carolina, United States of America; 2 Department of Chemistry, Georgetown University, NW, Washington DC, United States of America; 3 Department of Biochemistry and Molecular & Cellular Biology, Georgetown University, NW, Washington DC, United States of America; 4 Center for Tropical and Emerging Global Diseases, University of Georgia, Athens, Georgia, United States of America; 5 Department of Cellular Biology, University of Georgia, Athens, Georgia, United States of America; 6 Department of Chemistry, Biology and Biotechnology, University of Perugia, Perugia, Italy; University of Geneva, SWITZERLAND

## Abstract

*Toxoplasma gondii* is an apicomplexan parasite with the ability to use foodborne, zoonotic, and congenital routes of transmission that causes severe disease in immunocompromised patients. The parasites harbor a lysosome-like organelle, termed the "Vacuolar Compartment/Plant-Like Vacuole" (VAC/PLV), which plays an important role in maintaining the lytic cycle and virulence of *T*. *gondii*. The VAC supplies proteolytic enzymes that contribute to the maturation of invasion effectors and that digest autophagosomes and endocytosed host proteins. Previous work identified a *T*. *gondii* ortholog of the *Plasmodium falciparum* chloroquine resistance transporter (PfCRT) that localized to the VAC. Here, we show that TgCRT is a membrane transporter that is functionally similar to PfCRT. We also genetically ablate *TgCRT* and reveal that the TgCRT protein plays a key role in maintaining the integrity of the parasite’s endolysosomal system by controlling morphology of the VAC. When TgCRT is absent, the VAC dramatically increases in volume by ~15-fold and overlaps with adjacent endosome-like compartments. Presumably to reduce aberrant swelling, transcription and translation of endolysosomal proteases are decreased in Δ*TgCRT* parasites. Expression of subtilisin protease 1 is significantly reduced, which impedes trimming of microneme proteins, and significantly decreases parasite invasion. Chemical or genetic inhibition of proteolysis within the VAC reverses these effects, reducing VAC size and partially restoring integrity of the endolysosomal system, microneme protein trimming, and invasion. Taken together, these findings reveal for the first time a physiological role of TgCRT in substrate transport that impacts VAC volume and the integrity of the endolysosomal system in *T*. *gondii*.

## Author summary

*Toxoplasma gondii* is an obligate intracellular protozoan parasite that belongs to the phylum Apicomplexa and that infects virtually all warm-blooded organisms. Approximately one-third of the human population is infected with *Toxoplasma*. *Toxoplasma* invades host cells using processed invasion effectors. A lysosome-like organelle (VAC) is involved in refining these invasion effectors to reach their final forms. A *T*. *gondii* ortholog of the malarial chloroquine resistance transporter protein (TgCRT) was found to be localized to the VAC membrane. Although the mutated version of the malarial chloroquine resistance transporter (PfCRT) has been shown to confer resistance to chloroquine treatment, its physiologic function remains poorly understood. Comparison between the related PfCRT and TgCRT facilitates definition of the physiologic role of CRT proteins. Here, we report that TgCRT plays a key role in affecting the integrity and proteolytic activity of the VAC and adjacent organelles, the secretion of invasion effectors, and parasite invasion and virulence. To relieve osmotic stress caused by VAC swelling when TgCRT is deleted, parasites repress proteolysis within this organelle to decrease solute accumulation, which then has secondary effects on parasite invasion. Our findings highlight a common function for PfCRT and TgCRT in mediating small solute transport to affect apicomplexan parasite vacuolar size and function.

## Introduction

*Toxoplasma gondii* uses polypeptide invasion factors to efficiently invade host cells. These proteins are stored in two unique sets of organelles in *Toxoplasma* parasites, micronemes and rhoptries. Microneme proteins undergo a series of proteolytic cleavage steps within the parasite's endosomal system, followed by further trimming and intramembrane cleavage on the parasite surface [1,2]. Proper maturation and secretion of microneme proteins are crucial for efficient invasion of parasites [3–5].

Microneme protein maturation is conducted by several proteases. During intracellular trafficking, microneme proteins are first cleaved by aspartyl protease 3 (TgASP3) in a post-Golgi compartment [5]. A cathepsin L-like protease (TgCPL) was also shown to help process some microneme proteins in the endosome-like compartment (ELC) of the parasite [4]. The mature proteins then pass through the micronemes and undergo further trimming and intramembrane cleavage on the parasite surface. More specifically, a subtilisin ortholog, TgSUB1, was shown to trim some microneme proteins including microneme protein 2 (TgMIC2) and TgMIC2-associated protein (TgM2AP) on the parasite surface [3]. Subsequently, an integral membrane protease, rhomboid 4 (TgROM4), intramembranously cleaves transmembrane microneme proteins to release them from the cell surface. TgROM4 substrates include TgMIC2 and apical membrane antigen 1 (TgAMA1) [5–8]. Overall, precise control of proteolytic activities within the parasite’s endosomal system and on the plasma membrane is critical for processing parasite invasion effectors.

Among these proteases, TgCPL and TgSUB1 are both localized to, or transit through, the parasite’s endolysosomal system. TgCPL is located in a lysosome-like organelle, termed Vacuolar Compartment (VAC) or Plant-Like Vacuole (PLV) (hereafter referred to as VAC) in *Toxoplasma* parasites [4,9,10]. Our previous studies showed that the genetic ablation of *TgCPL* causes defects in parasite invasion and acute virulence [4,11]. TgCPL becomes activated in the VAC and a portion of TgCPL is delivered to the juxtaposed ELC for maturation [4]. TgSUB1 is a micronemal protease and contains a GPI anchor necessary for membrane association [3]. TgSUB1 was shown to be activated in a post-ER compartment and to transit through the parasite’s endolysosomal system before trafficking to micronemes [12]. Deletion of *TgSUB1* leads to inefficient trimming of microneme proteins on the parasite surface, thereby resulting in defects in invasion and virulence [3]. Hence, maintaining integrity of the parasites’ endolysosomal system is critical for regulating the distribution and activity of endolysosomal proteases.

In addition to VAC dysfunction resulting in reduced invasion, replication, and virulence [4,11], parasites with impaired VAC proteolytic function are unable to turn over autophagosomes during chronic infection and thereby cannot survive in host brain tissues [13]. Despite its importance, the VAC has not been well characterized. Only a few proteins have been localized to the VAC/PLV [4,10,14–17]. The VAC exists as a prominent intact organelle during initial infection and subsequently fragments during intracellular replication, based on staining of TgCPL, a major luminal protease in the VAC [4]. It is unknown how parasites regulate these and other morphological changes that occur within the endolysosomal system. In a previous study, *TgCRT* expression was knocked down in Type I *Toxoplasma* parasites using a tetracycline-inducible system [15], and VAC swelling was observed, suggesting that the function of TgCRT is necessary to maintain normal VAC volume. Fitness defects were also seen in the *TgCRT* knockdown strain [15]. However, a detailed characterization of how an altered VAC affects different steps of parasite intracellular growth is still missing and the corresponding molecular mechanisms underlying the phenotypes are not understood.

Interestingly, the swollen VAC phenotype for the *TgCRT* knockdown mirrors the enlarged digestive vacuole (DV) phenotype for chloroquine-resistant (CQR) *Plasmodium falciparum* expressing CQR-associated mutant PfCRT [18]. More recently, an L272F PfCRT mutation, along with CQR-conferring mutations, was found to increase DV volume by an additional 1–2 μm^3^ [19]. *In vitro* assays using purified recombinant PfCRT, reconstituted in proteoliposomes, suggest that PfCRT transports aminoquinoline drugs, basic amino acids, and perhaps oligopeptides, likely in an electrochemically coupled fashion [20,21]. With respect to drug transport, PfCRT expressed within CQR *P*. *falciparum* appears to exhibit higher chloroquine (CQ) transport efficiency relative to PfCRTs found in chloroquine-sensitive (CQS) strains [20–22]. These findings suggest that the PfCRT mediates the transport of key osmolytes from the *P*. *falciparum* DV. Unfortunately, the inability to successfully ablate the *PfCRT* gene [23] limits additional analysis of function *in vivo*.

Here, we successfully delete the *TgCRT* gene in a Type I *Toxoplasma* parasite strain by double crossover homologous recombination. The resulting mutant, Δ*crt*, displayed a severely swollen VAC and aberrant colocalization of markers for the VAC and ELC. Surprisingly, this aberrant organellar organization is associated with down-regulated transcription and translation of several proteases residing in the parasite’s endolysosomal system, altering microneme secretion and resulting in defective parasite invasion and acute virulence. We also engineer successful overexpression of wild type TgCRT constructs in yeast and show that the protein mediates CQ transport. Collectively, these findings determine a novel role for maintaining endolysosomal integrity, suggest functional similarities for TgCRT and PfCRT proteins, and provide a new model system for analyzing the function of apicomplexan CRT proteins.

## Results

### *TgCRT*-deficient parasites lose endolysosomal system integrity due to altered VAC morphology

Previous studies have localized TgCRT in the VAC by gene epitope-tagging and immunofluorescence microscopy, and utilized an anhydrotetracycline-regulated system to reduce levels of expression of *TgCRT* in a Type I *Toxoplasma* RH strain. These results revealed that TgCRT is involved in volume control of the VAC [15]. However, incomplete depletion of *TgCRT* limits further characterization of its function. Additionally, lack of detailed phenotypic characterization restricts our understanding of how the swollen VAC affects parasite fitness and virulence. Here, we adopted a genetically tractable RH-derived strain, termed RHΔ*ku80* (hereafter referred to as WT), to produce a complete *TgCRT* knockout (refer to **S1 Text, Table 1, and S1A and S1B Fig** for more details). The RHΔ*ku80* strain lacks non-homologous end-joining DNA repair, thus enhancing homology-dependent DNA recombination [24]. Due to the increased homologous recombination efficiency, this strain has been widely used as a wild type *Toxoplasma* strain.

**Table 1:** Parasite strains used in this study.

Name	Genetic background	Comments
WT	RHΔ*ku80*Δ*hxg*	Requested from the Carruthers Lab, not generated in this study
Δ*crt*	RHΔ*ku80*Δ*hxg*Δ*crt*	*TgCRT* was deleted by double-crossover homologous recombination
Δ*crtCRT*	RHΔ*ku80*Δ*hxg*Δ*crt*::*TgCRT-mCherry-3xmyc*	Ectopic expression of a C-terminally epitope-tagged TgCRT in Δ*crt* for complementation
Δ*crtCRT*^*T369K*^	RHΔ*ku80*Δ*hxg*Δ*crt*::*TgCRT*^*T369K*^*-mCherry-3xmyc*	Ectopic expression of a C-terminally epitope-tagged TgCRT mutant in Δ*crt* for complementation. The original threonine at position 369 within TgCRT was changed to lysine by site-directed mutagenesis.
Δ*crt*Δ*cpb*	RHΔku80ΔhxgΔ*crt*Δ*cpb*	The entire *TgCPB* gene was ablated by CRISPR-Cas9 based genome editing technique
Δ*crt*Δ*cpl*	RHΔku80ΔhxgΔ*crt*Δ*cpl*	The entire *TgCPL* gene was ablated by CRISPR-Cas9 based genome editing technique
Δ*crt*Δ*sub1*	RHΔku80ΔhxgΔ*crt*Δ*sub1*	The entire *TgSUB1* gene was ablated by CRISPR-Cas9 based genome editing technique
WT::*nLuc*	RHΔ*ku80*Δ*hxg*::*nLuc*	Expressed NanoLuc luciferase in WT parasites
Δ*crt*::*nLuc*	RHΔ*ku80*Δ*hxg*Δ*crt*::*nLuc*	Expressed NanoLuc luciferase in Δ*crt* parasites
Δ*crtCRT*::*nLuc*	RHΔ*ku80*Δ*hxg*Δ*crt*::*TgCRT-mCherry-3xmyc*::*nLuc*	Expressed NanoLuc luciferase in Δ*crtCRT* parasites
TgAMN-3xHA	RHΔ*ku80*Δ*hxgTgAMN-3xHA*	*TgAMN* gene was endogenously tagged with a 3xHA epitope at its 3'-end by CRISPR-Cas9 based genome editing technique
TgSCP-3xmyc	RHΔ*ku80*Δ*hxgTgSCP-3xmyc*	*TgSCP* gene was endogenously tagged with a 3xmyc epitope at its 3'-end by single-crossover recombination
WT::*PHL2*	RHΔ*ku80*Δ*hxg*::*PHL2*	Expressed pHluorin2 in the cytoplasm of WT parasites
Δ*crt*::*PHL2*	RHΔ*ku80*Δ*hxg*Δ*crt*::*PHL2*	Expressed pHluorin2 in the cytoplasm of Δ*crt* parasites
Δ*crtCRT*::*PHL2*	RHΔ*ku80*Δ*hxg*Δ*crt*::*TgCRT-mCherry-3xmyc*::*PHL2*	Expressed pHluorin2 in the cytoplasm of Δ*crtCRT* parasites
Δ*vp1*	RHΔ*ku80*Δ*hxg*Δ*vp1*	Requested from the Moreno Lab, not generated in this study
Δ*vp1VP1*	RHΔ*ku80*Δ*hxg*Δ*vp1VP1*	Requested from the Moreno Lab, not generated in this study

**Fig 1:**
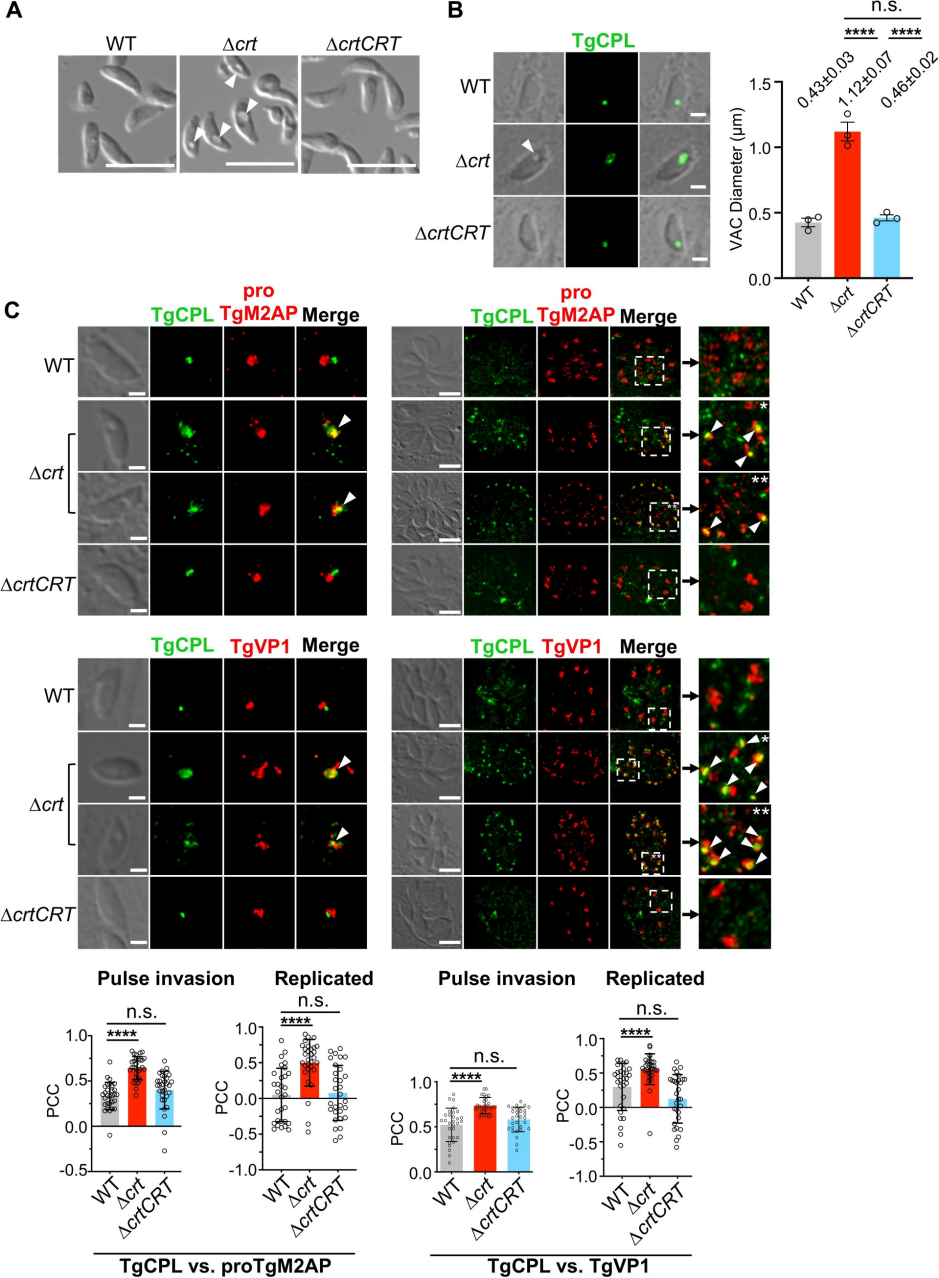
The *TgCRT*-deficient parasites displayed a swollen vacuole and a disrupted endolysosomal system. (A) Extracellular Δ*crt* parasites showed an enlarged concave subcellular structure, indicated by the arrow, under the differential interference contrast (DIC) microscopy. Scale bar = 5 μm. (B) The swollen subcellular structure indicated by the arrow was also observed in pulse-invaded *Δcrt* parasites and colocalized with a major luminal peptidase of the VAC, cathepsin L-like protease (TgCPL). The TgCPL staining was used to assess the morphology of the VAC. The mean VAC size ± standard error of mean (SEM) was calculated for three independent measurements and is listed in the figure. Statistical significance was determined using unpaired two-tailed Student’s *t*-test. Scale bar = 2 μm. (C) In pulse-invaded parasites, the TgCPL (VAC marker) and proTgM2AP or TgVP1 (ELC markers) staining were juxtaposed in the WT and Δ*crtCRT* strains, but co-localized in the Δ*crt* strain. During replication, the VAC in WT and Δ*crtCRT* parasites became fragmented. However, the aberrant overlap between the VAC and ELC significantly decreased the extent of VAC fragmentation in the Δ*crt* mutant. TgCPL staining presented as one or more larger and intensely stained puncta, which co-localized with proTgM2AP and TgVP1 (indicated by arrows in the insets). The scale bars in the images of pulse invaded and replicated parasites are 2 μm and 5 μm, respectively. The colocalization of the TgCPL with proTgM2AP or TgVP1 was analyzed by a Coloc2 colocalization analysis software. The Pearson’s correlation coefficient (PCC) of both VAC and ELC staining was measured within 10 individual parasites for each replicate in a total of three replicates and presented as mean ± standard deviation (SD). Statistical significance was determined using unpaired two-tailed Student’s *t*-test. ****, *p*<0.0001; n.s., not significant.

Upon generating RHΔ*ku80*Δ*crt*, we observed that purified extracellular Δ*crt* parasites exhibited large “concave” subcellular structures under differential interference contrast (DIC) microscopy, whereas WT and Δ*crtCRT* strains did not display this phenotype **(Fig 1A)**. This subcellular structure was also observed in pulse-invaded Δ*crt* parasites **(Fig 1B)**. To identify the swollen structures, we stained the WT, Δ*crt*, and Δ*crtCRT* parasites with anti-TgCPL antibodies. TgCPL is a major luminal endoprotease in the VAC of *Toxoplasma* [4,25]. Immunofluorescence microscopy showed that TgCPL staining co-localized with concave subcellular structures in Δ*crt* **(Fig 1B)**. The TgCPL staining in Δ*crt* was larger than that in WT and Δ*crtCRT* parasites, indicating that the VAC becomes swollen when TgCRT is absent. We quantified the VAC sizes based on TgCPL staining as described previously [13,15]. VAC diameter for the Δ*crt* parasites (1.12 ± 0.07 μm) is approximately 2.6-fold larger than for WT parasites (0.43 ± 0.03 μm), while the Δ*crtCRT* (0.46 ± 0.02 μm) VAC was similar to that measured for WT parasites **(Fig 1B)**. If we assume the VAC is approximately spherical, then the Δ*crt* parasite VAC is approximately 15-fold larger than the WT VAC. In contrast to pulse-invaded parasites, the swollen concave structure was not observed in replicated Δ*crt* parasites **(Fig 1C)**. However, TgCPL staining showed differences between WT and Δ*crt* parasites **(Fig 1C)**. For WT, the VAC displayed dynamic structures during replication that appeared as small fragmented puncta upon TgCPL staining [4]. However, TgCPL staining revealed fewer small fragmented punctate structures in replicating Δ*crt* parasites and instead featured one or more larger and intensely stained puncta **(Fig 1C)**. Overall, we found that loss of TgCRT severely alters the morphology of the VAC in pulse-invaded as well as replicating *Toxoplasma*.

The VAC is a lysosome-like organelle, participating in the parasite’s endolysosomal system. It provides an environment for maturation of TgCPL and delivers activated TgCPL to its adjacent endosome-like compartment (ELC) to assist in processing microneme proteins required for parasite invasion [4]. It also serves as a digestive organelle to degrade endocytosed proteins [4,11,26]. We hypothesized that the dramatic swelling of the VAC might affect the integrity of the parasite’s endolysosomal system. We stained WT, Δ*crt*, and Δ*crtCRT* parasites with antibodies recognizing markers of the VAC (anti-TgCPL) and of the ELC (anti-proTgM2AP or TgVP1) [4,10], and captured a series of deconvolved Z-stack images for individual co-staining. In pulse-invaded parasites, the VAC and ELC displayed distinct subcellular staining in WT and Δ*crtCRT* strains, whereas in Δ*crt* parasites both markers partially co-localized **(Fig 1C).** Similarly, the non-fragmented TgCPL puncta in replicating Δ*crt* parasites also showed partial colocalization with both proTgM2AP and TgVP1 **(Fig 1C)**. We quantified the colocalization of TgCPL with proTgM2AP and TgCPL with TgVP1 by measuring their Pearson’s correlation coefficient (PCC). Our analysis revealed that TgCPL showed significantly higher colocalization with proTgM2AP or TgVP1 in Δ*crt* parasites than WT and Δ*crtCRT* strains in both stages of pulse invasion and replication **(Fig 1C)**. A sodium/proton exchanger, named TgNHE3, was previously identified in the ELC [27]. We also co-stained WT, Δ*crt*, and Δ*crtCRT* strains with anti-TgCPL and anti-TgNHE3 antibodies, and did not observe partial colocalization in Δ*crt* mutant **(S2 Fig)**. These results mirror a previous observation that the ingested host proteins in the *TgCPL*-deficient parasites overlapped with proTgM2AP to a greater extent than TgNHE3 during their trafficking through the parasite’s endolysosomal system [26], suggesting that the TgNHE3 occupies a distinct subdomain of the ELC. Overall, our findings suggest that altered VAC morphology due to the absence of TgCRT affects the integrity of the parasite’s endolysosomal system.

### RHΔ*ku80*Δ*crt* shows reduced invasion and acute virulence

**Fig 2:**
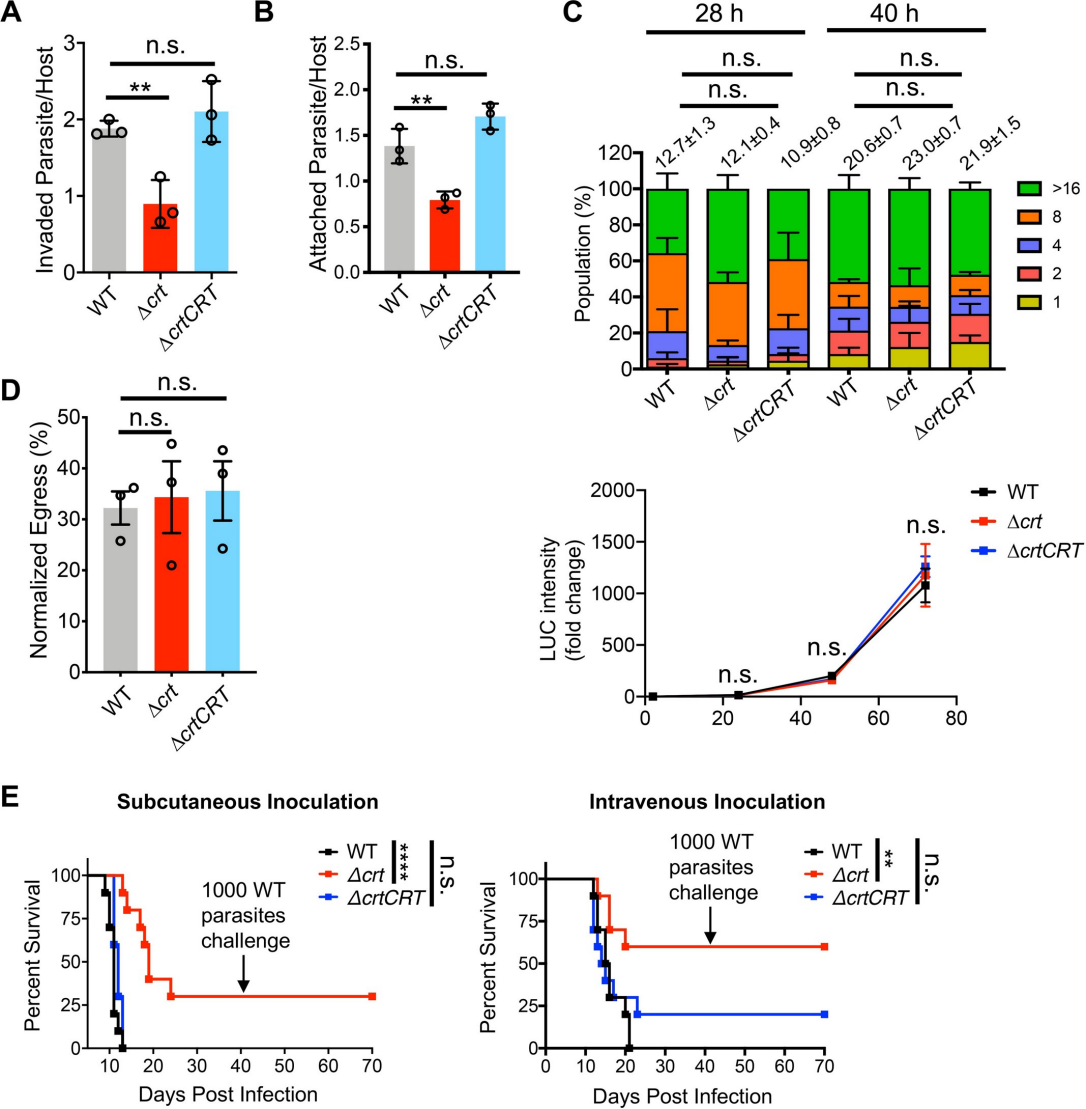
Parasite invasion and acute virulence were reduced in the Δ*crt* parasites. (A) At 30 min post-infection, the Δ*crt* parasites showed 0.90 ± 0.31 (mean ± SD) parasites per host cell, which is approximately a ~50% reduction in invasion compared to the WT (1.88 ± 0.10) and Δ*crtCRT* (2.10 ± 0.40) strains. Six fields of view were counted per strain per replicate. The assay was performed in triplicate. (B) The Δ*crt* mutant exhibited ~50% reduction in attachment compared to WT and Δ*crtCRT* strains. Eight view fields were captured and quantified per strain per replicate. The assay was performed in triplicate. (C) The Δ*crt* displayed comparable replication rates as WT and Δ*crtCRT* parasites. Both fluorescence- and luminescence-based replication assays showed similar results. The average number of parasites per parasitophorous vacuole derived from the fluorescence-based replication assay was also calculated and listed above the plots as mean ± SD. (D) A lactate dehydrogenase release assay was used to measure the egress efficiency of parasites. No differences were observed in parasite egress between WT and Δ*crt* parasites. Statistical significance in the assays listed in panel A through D was determined by using unpaired two-tailed Student’s *t*-test. (E) The acute virulence of *TgCRT*-deficient parasites was evaluated in a murine model via subcutaneous and intravenous infections. One hundred parasites from each strain were used to infect outbred CD-1 mice (n = 10 mice for each strain). The mortality of the mice was monitored for 30 days. Seroconversion of the surviving mice was evaluated by ELISA to confirm successful infection. Additionally, the surviving mice were allowed to rest for 10 days before subsequent challenge with 1,000 WT parasites by subcutaneous inoculation. The Δ*crt* mutant exhibited reduced acute virulence compared to the WT and Δ*crtCRT* strains and conferred immunological protection in the surviving mice. Data were recorded and are presented using the Kaplan-Meier plot. Statistical analysis was performed using the Log-rank (Mantel-Cox) test. For all statistical significance calculation, **, *p*<0.01; ****, *p*<0.0001; n.s, not significant.

*Toxoplasma* utilizes exocytosis and endocytosis via endolysosomes to release invasion effectors, and to ingest host proteins during intracellular growth, respectively [11,28–30]. We therefore characterized invasion, replication, and egress for the Δ*crt* strain. First, we measured the invasion efficiency of parasites at 30–120 min post-infection. At 30 min post-infection, the Δ*crt* mutant showed ~50% reduction in invasion compared to WT and Δ*crtCRT* **(Fig 2A)**. Differences in invasion efficiency between WT and Δ*crt* were reduced by ~20% at 60 min post-infection, and were not seen at 120 min post-infection **(S3A Fig)**, suggesting that Δ*crt* parasites have slower invasion kinetics relative to the WT strain. To further understand the basis of this invasion deficiency, we compared parasite attachment to host cells using previously published methods [3,31]. We found that the Δ*crt* mutant showed ~50% reduction in host cell attachment compared to WT and Δ*crtCRT* strains **(Fig 2B)**. In contrast, we observed no significant differences in both gliding distance and types **(S3B Fig)**. Second, we used immunofluorescence microscopy to quantify parasite replication. Infected cells were stained with DAPI and anti-TgGRA7 antibodies to define individual parasite nuclei and parasitophorous vacuolar (PV) membranes, respectively. The average number of parasites per PV was calculated for each strain to compare replication rates. There were no statistical differences in parasite replication between WT and Δ*crt* parasites at 28 and 40 h post-infection **(Fig 2C)**. We also introduced NanoLuc luciferase into WT, Δ*crt*, and Δ*crtCRT* parasites, and measured the fold-change of luciferase activity for 72 h post-infection to calculate relative growth rates. Similarly, we did not observe growth differences between WT and Δ*crt* at 24, 48, and 72 h post-infection **(Fig 2C)**. Third, the egress efficiency of each strain was determined by a lactate dehydrogenase release-based assay. The parasites were incubated with 0.5 mM Zaprinast for 5 min to induce egress. The egressed parasites disrupt host cell membranes to release lactate dehydrogenase, which is subsequently quantified to extrapolate to the number of egressed PVs. We did not observe egress defects in the *TgCRT*-deficient parasites **(Fig 2D)**. Last, we determined the acute virulence of Δ*crt* parasites in a murine model. Outbred CD-1 mice were infected with a subcutaneous or intravenous inoculum of 100 WT, Δ*crt*, or Δ*crtCRT* parasites. Thirty percent of mice infected with the Δ*crt* mutant survived when mice were infected subcutaneously, whereas WT and Δ*crtCRT* infections led to quantitative mortality at 10–12 days post-infection **(Fig 2E)**. Mice receiving WT parasites by intravenous inoculation showed mortality starting at 13 days post-infection and all expired at 20 days. The Δ*crt* and Δ*crtCRT* parasites caused death in 40% and 80% of infected mice, respectively **(Fig 2E)**. Statistical analysis showed that mice infected with WT and Δ*crt* parasites have significant difference in their survival time. Seroconversion of the surviving mice was confirmed by ELISA. We also challenged surviving Δ*crt* mice with 1000 WT parasites by subcutaneous injection and did not observe lethality after 30 days post-challenge. These findings indicate that the pre-inoculation of Δ*crt* parasites conferred immunological protection against subsequent acute toxoplasmosis. Given the hyper-virulent nature of Type I *Toxoplasma* strain in a murine model, the Δ*crt* mutant dramatically lost its acute virulence compared to WT parasites. Collectively, our findings revealed that *Toxoplasma* parasites require the TgCRT protein for optimal invasion and acute virulence but not for replication and egress.

### Δ*crt* parasites show impaired microneme secretion

**Fig 3:**
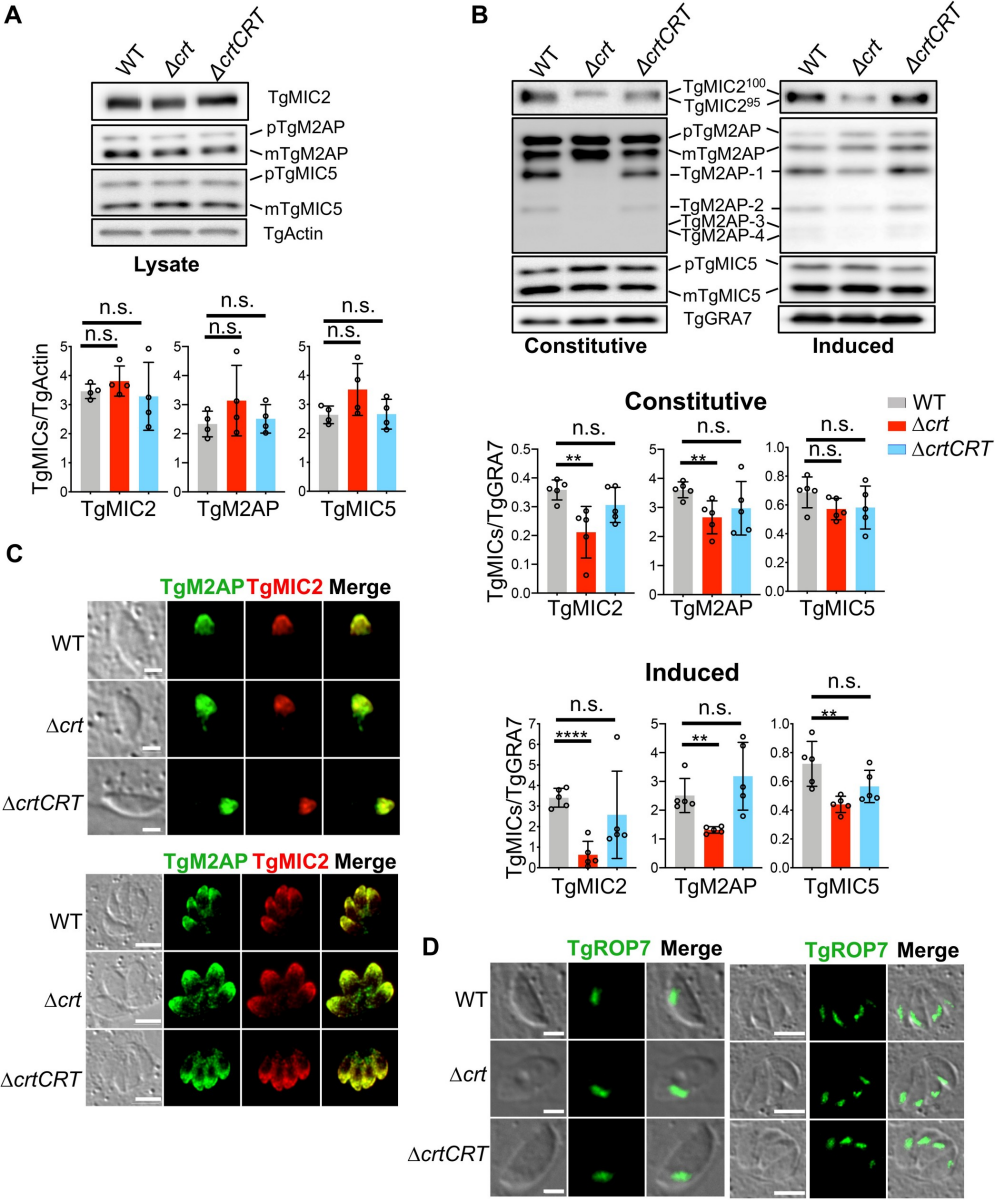
The deletion of *TgCRT* altered microneme secretion, without affecting the microneme steady abundance, intracellular trafficking, or intramembrane cleavage on the parasite surface. (A) The steady-state level of microneme proteins was not altered in the Δ*crt* parasites. Freshly lysed parasites were filter-purified, lysed, and subjected to SDS-PAGE electrophoresis and immunoblotting. The blots were probed with anti-TgMIC2, TgM2AP, and TgMIC5 antibodies, along with anti-TgActin as a loading control. Individual microneme proteins were normalized against the corresponding TgActin to quantify their steady-state expression. Four independent preparations and probing of parasite lysates were conducted in this assay. (B) Δ*crt* parasites secreted fewer microneme proteins than WT and Δ*crtCRT* parasites, and altered the microneme secretion patterns. Freshly filter-purified parasites were incubated in medium at 37°C for 30 min to generate the constitutive ESAs, or were treated with 1% (v/v) ethanol in medium to produce induced ESAs. The ESA fractions were separated and probed with anti-TgMIC2, TgM2AP, and TgMIC5 antibodies for quantification of the secreted forms of these microneme proteins. The ESA fractions were also probed with anti-TgGRA7 antibody, a dense granule protein, as a loading control. Five independent preparations and probing of both constitutive and induced ESAs were performed in this assay. (C) Pulse-invaded and replicated parasites were stained with anti-TgMIC2 and anti-TgM2AP antibodies to examine their intracellular trafficking. No defects were detected in their intracellular trafficking. (D) Pulse-invaded and replicated parasites were also stained with anti-TgROP7 to examine the morphology of the rhoptry and intracellular trafficking of TgROP7. The rhoptries maintained similar morphology and trafficking patterns among WT, Δ*crt*, and Δ*crtCRT* strains. The scale bars in the images of pulse invaded and extracellular parasites are 2 μm, and the scale bars in the images of replicated parasites are 5 μm. The statistical significance among the steady expression and secretion of microneme proteins in WT, Δ*crt*, and Δ*crtCRT* strains was determined using unpaired two-tailed Student’s *t*-test. **, *p*<0.01; ****, *p*<0.0001; n.s., not significant.

During infection, *Toxoplasma* parasites sequentially secrete proteins to facilitate host invasion. Microneme proteins are the first to be secreted. These proteins traffic through the parasite’s endolysosomal system and undergo intracellular maturation before being trimmed and released from the parasite surface by intramembrane cleavage [3–8,32]. To test which step(s) is affected in the Δ*crt* parasites, we probed cell lysates and excretory secretory antigen fractions (ESAs) of each strain with anti-TgMIC2, anti-TgM2AP, and anti-TgMIC5 by immunoblotting to measure abundances and secretion patterns. The migration patterns of these three microneme proteins in cell lysates were similar among the strains **(Fig 3A)**. The abundances of the individual microneme proteins were normalized against the protein level of the *Toxoplasma* actin protein by densitometry and plotted for quantification. All three strains showed comparable steady-state abundances of these proteins **(Fig 3A)**. To further evaluate abundances of secreted microneme proteins, we probed constitutive and induced ESAs with the same antibodies. The constitutive and induced ESAs were generated by incubating purified parasites in D10 medium (DMEM medium supplemented with 10% (v/v) cosmic calf serum) for 30 min at 37°C or D10 medium supplemented with 1% (v/v) ethanol for 2 min at 37°C, respectively. In the ESAs secreted by WT parasites, TgMIC2 exhibited two bands migrating at 100 kDa and 95 kDa, while TgM2AP showed 4 proteolytically processed polypeptides along with pro- and mature forms. However, TgMIC2 only existed as a 100 kDa band in Δ*crt* parasites. Furthermore, mature TgM2AP was not processed in the constitutive ESAs of the Δ*crt* strain and showed significantly reduced processing in the induced ESAs of Δ*crt* parasites **(Fig 3B)**. The secreted TgMIC5 protein displayed similar migration patterns among these strains **(Fig 3B)**. Secretion of these microneme proteins was also quantified by normalizing the relative abundances of the proteins against the protein level of secreted TgGRA7, a dense granule protein. The secretion of TgMIC2, TgM2AP, and TgMIC5 were reduced by ~80%, 50%, and 40%, respectively, in the induced ESAs of Δ*crt* parasites, compared to the WT strain. The differences in the amount of microneme secretion were less significant in the constitutive ESAs. Secretion of TgMIC2 and TgM2AP was decreased by ~40% and 25%, respectively, in Δ*crt* parasites compared to the WT strain, whereas TgMIC5 did not show a difference **(Fig 3B)**. Lower ESA secretion induced by ethanol in the Δ*crt* mutant suggests that Δ*crt* parasites are deficient in rapid release of certain proteins from the micronemes.

To examine whether the abnormal secretion of microneme proteins alters their intracellular trafficking patterns, we stained pulse-invaded and replicated parasites with TgMIC2 and TgM2AP antibodies. Both microneme proteins trafficked to the apical end of the parasites and showed normal staining patterns **(Fig 3C)**. Prior to secretion, some transmembrane microneme proteins are released via proteolytic cleavage by the intramembrane rhomboid protease TgROM4. Deletion of *TgROM4* leads to retention of some microneme proteins on the parasite’s plasma membrane, such as TgMIC2 and TgAMA1 (*Toxoplasma* apical membrane antigen 1) [6–8,32]. To test whether the aberrant endolysosomal system alters the retention of microneme proteins on the surface of parasites, we stained purified, non-permeabilized extracellular parasites with anti-TgMIC2 antibodies. Immunofluorescence microscopy did not reveal excess TgMIC2 on the plasma membrane of Δ*crt* parasites **(S4 Fig)**, suggesting that the reduced secretion of microneme proteins is not due to their inefficient intramembrane cleavage on the parasite’s plasma membrane.

The endosome-like compartment is involved not only in the trafficking of microneme proteins, but also rhoptry contents [33]. We stained pulse-invaded and replicated parasites with anti-TgROP7 antibodies to examine the trafficking of rhoptry proteins and the morphology of rhoptries. TgROP7 staining revealed typical rhoptry patterns located at the apical end of the parasites (**Fig 3D**), excluding the possibility of aberrant trafficking of rhoptry contents and possible defects in rhoptry biogenesis. Taken together, our data suggest that the invasion defects for Δ*crt* parasites are caused by incomplete trimming and consequent inefficient secretion of microneme proteins at the parasite’s plasma membrane, but not by altered intracellular maturation, trafficking, or intramembrane cleavage of microneme proteins, nor by altered rhoptry morphology.

### TgSUB1 transcript and protein levels are decreased for Δ*crt* parasites

**Fig 4:**
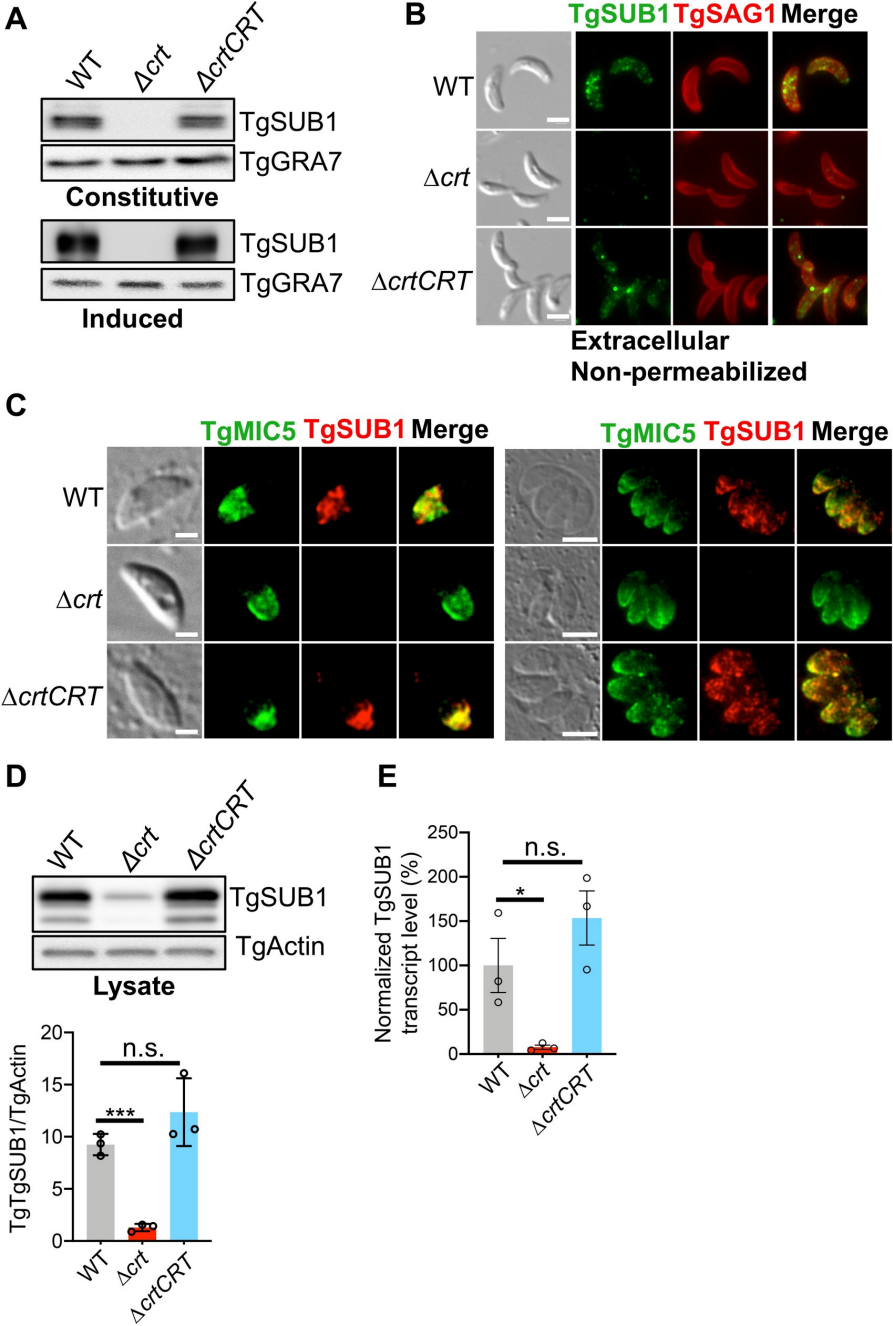
Transcript and protein levels of a subtilisin-related protease, TgSUB1, were reduced in Δ*crt* parasites. (A) The abundance of secreted TgSUB1 in the constitutive and induced ESAs was measured by immunoblotting. There was no detectable TgSUB1 in the constitutive and induced ESAs secreted by Δ*crt* parasites via immunoblotting. (B) The abundance of plasma membrane-anchored TgSUB1 was measured by probing non-permeabilized parasites with antibodies recognizing TgSUB1. The Δ*crt* mutant significantly reduced TgSUB1 on its plasma membrane. (C) The intracellular TgSUB1 expression level was significantly reduced in Δ*crt* as shown by immunofluorescence microscopy. TgSUB1 was identified as a microneme protein. TgSUB1 and TgMIC5 were both found to localize in the micronemes of pulse-invaded and replicated WT and Δ*crtCRT* parasites. However, TgSUB1 signal was not detected in the Δ*crt* parasites. (D) The cell lysates of the Δ*crt* parasites showed that the steady level of TgSUB1 was reduced by ~85% in the Δ*crt* strain. (E) The transcript level of TgSUB1 was quantified by quantitative PCR. It was also decreased by approximately 90% in the Δ*crt* mutant. All assays listed in this figure were repeated in triplicate. Statistical significance was calculated using unpaired two-tailed Student’s *t-*test: *, *p*<0.05; ***, *p*<0.001; n.s., not significant.

The inefficient proteolytic processing of TgMIC2 and TgM2AP in Δ*crt* ESAs led us to investigate whether these phenotypic observations were caused by the abnormal expression patterns or subcellular trafficking of *Toxoplasma* subtilisin 1 (TgSUB1) in Δ*crt* parasites. A previous publication reported that parasites lacking TgSUB1 showed defective trimming of secreted microneme proteins, such as TgMIC2 and TgM2AP [3], which we noted resemble the altered patterns of TgMIC2 and TgM2AP products observed for the Δ*crt* mutant. Therefore, we quantified secreted TgSUB1 in both constitutive and induced ESAs by probing them with an anti-SUB1 antibody, previously found to specifically react against TgSUB1 and PfSUB1 [34]. Immunoblotting analysis revealed that there was no detectable TgSUB1 in the ESAs of Δ*crt* parasites **(Fig 4A)**. TgSUB1 stays on the plasma membrane via its GPI-anchor prior to its release by self-shedding [12,35]. Given that there was no detectable TgSUB1 in the ESAs of the Δ*crt* strain, we measured the abundance of TgSUB1 on the parasite surface by immunofluorescence microscopy to assess if the Δ*crt* retained TgSUB1 on the plasma membrane. We stained non-permeabilized extracellular parasites with anti-SUB1 to evaluate the amount of surface-anchored TgSUB1. Similarly, no detectable TgSUB1 was observed on the plasma membrane of Δ*crt* parasites **(Fig 4B)**. These data suggest that there is lower expression of TgSUB1 on the surface of Δ*crt* parasites.

To further dissect the basis for reduced TgSUB1 on the surface, we tested two possibilities: 1) TgSUB1 traffics aberrantly within the parasite to prevent its delivery to parasite surface, and 2) the expression level of TgSUB1 is reduced. TgSUB1 is a microneme protein that also traffics through the parasite’s endolysosomal system [3,12]. The aberrant endolysosomal system in Δ*crt* parasites potentially alters intracellular trafficking and/or maturation of TgSUB1 that then reduces its expression. To test these two possibilities, first, we stained pulse-invaded and replicated parasites with anti-SUB1 to examine TgSUB1 intracellular trafficking patterns. TgMIC5 localization was used as a reference for typical expected microneme staining. Surprisingly, we observed much less TgSUB1 staining in Δ*crt* parasites compared to the WT strain **(Fig 4C)**. Next, we quantified abundance of TgSUB1 in parasite cell lysates and found that TgSUB1 was decreased by approximately 85% in Δ*crt* parasites compared to WT parasites **(Fig 4D)**. To further understand how TgSUB1 expression is suppressed in the Δ*crt* mutant, we performed qPCR to measure *TgSUB1* mRNA for WT, Δ*crt*, and Δ*crtCRT* parasites. *TgSUB1* transcript was reduced ~10-fold upon deletion of *TgCRT* **(Fig 4E)**. Collectively, our findings suggest that the arrested overlap of the VAC and ELC dramatically decreases the abundance of TgSUB1 protein, which then alters the proteolytic processing of normally secreted micronemal protein invasion effectors, thereby reducing invasion efficiency.

### VAC alterations reduce endolysosomal protease proteins and transcripts

**Fig 5:**
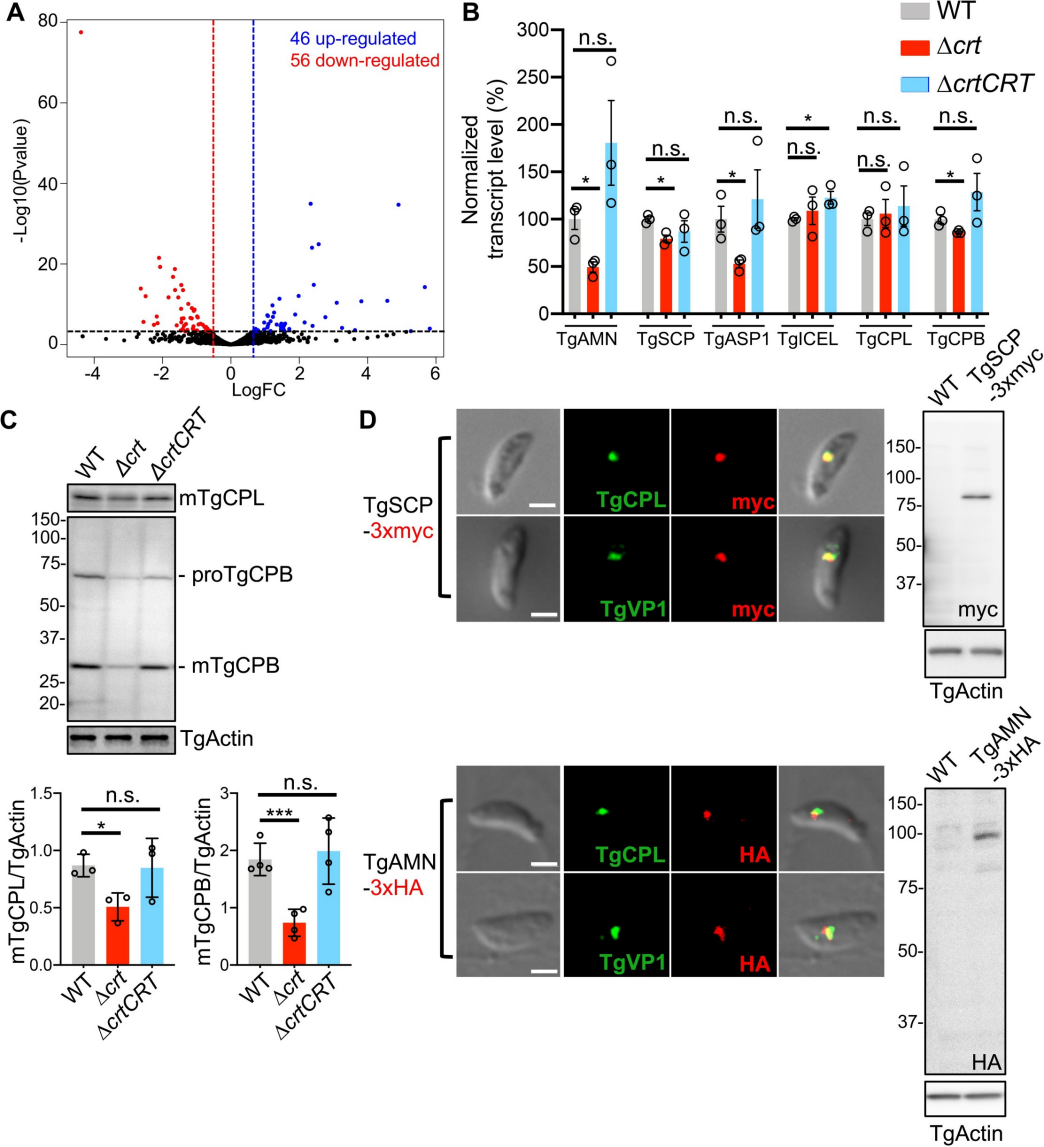
The transcript and protein abundances of several VAC-residing proteases were decreased in Δ*crt*. (A) RNA-Seq was performed in WT and Δ*crt* parasites. Each sample was sequenced in duplicate for statistical comparison. A volcano plot was used to summarize the genes with altered transcription greater than 1.5-fold and with statistical significance less than 0.05 in the Δ*crt* mutant relative to the WT strain. Forty-six and fifty-six genes labeled in the blue and red dots became up- and down-regulated in the Δ*crt* mutant, respectively. The blue and red dash lines represent the borderline of 1.5-fold change in gene transcripts, and the genes above the black dash line had *p* values of statistical significance below 0.05. (B) qPCR was used to validate 4 down-regulated proteases that were identified by RNA-Seq analysis, along with two known VAC proteases, TgCPL and TgCPB. *TgAMN*, *TgSCP*, *TgASP1*, and *TgCPB* displayed down-regulated transcription in Δ*crt* parasites compared to WT. The qPCR assay was repeated in three technical replicates per biological replicate in a total of three biological replicates. Data shown in the figure are represented as mean ± SEM. The fold change of individual genes was calculated using a ΔΔCT (cycle threshold) method for relative quantification by normalization against WT values using *TgActin* as a normalization control. The variation shown in WT parasites was determined by normalization of individual CT values per replicate against the input of total RNA. (C) The steady protein abundances of TgCPL and TgCPB were quantified in the lysates of parasites by immunoblotting. The protein levels of TgCPL and TgCPB in the Δ*crt* mutant were reduced by ~40% and 60%, respectively, compared to WT parasites. Immunoblotting assays were repeated in 3–4 replicates for statistical comparison. (D) TgSCP and TgAMN were endogenously tagged with 3xmyc and 3xHA, respectively, at their C-termini. The expression of the epitope-tagged proteins was confirmed by immunoblotting. The parasites were co-stained with antibodies recognizing their respective epitope tags as well as the VAC and ELC markers. Both TgSCP and TgAMN were localized in the VAC and ELC by immunofluorescence. Statistical significance was performed using unpaired two-tailed Student’s *t-*test. *, *p*<0.05; ***, *p*<0.001; n.s., not significant.

The swollen VAC and its aberrant overlap with the ELC in the Δ*crt* parasites could conceivably lead to altered gene transcription to assist in the adaptation of these parasites. We conducted transcriptome sequencing to detect global alterations in gene transcription for Δ*crt* parasites relative to WT. Differential gene expression analysis identified 102 genes whose transcript levels changed greater than 1.5-fold in the Δ*crt* strain. Forty-six and fifty-six genes had increased and reduced transcripts, respectively **(Fig 5A and S1 Table)**.

Four proteases were among the list of genes showing reduced transcripts in the Δ*crt* mutant, including a putative aminopeptidase N protein (TgAMN, TGGT1_221310), a putative Pro-Xaa serine carboxypeptidase (TgSCP, TGGT1_254010), aspartyl protease 1 (TgASP1, TGGT1_201840), and an ICE family protease-like protein (TgICEL, TGGT1_243298). We validated transcript levels for these proteases, as well as two known VAC luminal proteases (TgCPL and TgCPB), in WT, Δ*crt*, and Δ*crtCRT* strains by qPCR. The qPCR analysis showed that the transcript levels of TgAMN, TgSCP, TgASP1, and TgCPB were decreased by 50%, 20%, 47%, and 14%, respectively, in Δ*crt* parasites **(Fig 5B)**. Protein levels of TgCPL and TgCPB were quantified by immunoblotting and compared for WT, Δ*crt*, and Δ*crtCRT* parasites. Although TgCPL transcript levels did not differ, the abundance of TgCPL protein was decreased ~40% in the Δ*crt* mutant **(Fig 5C)**. *TgCPB* transcript levels were reduced in Δ*crt*, and both the pro- and mature forms of TgCPB protein were decreased relative to WT parasites **(Fig 5C)**. Densitometry analysis showed that the expression level of mature TgCPB was reduced by ~60% in the Δ*crt* parasites **(Fig 5C)**.

To determine the subcellular locations of the down-regulated proteases, we tagged endogenous TgAMN and TgSCP with 3xHA and 3xmyc epitope tags at their C-termini in WT parasites, respectively **(S5 Fig and Table 1)**. After drug-selection, we probed cell lysates with anti-HA and anti-myc antibodies, respectively, to test expression. Immunoblotting revealed that the observed molecular masses of both proteins were similar to the predicted sizes based on the primary sequences **(Fig 5D)**. Next, the tagged strains were co-stained with antibodies recognizing the epitope tags along with anti-TgCPL or anti-VP1 antibodies to determine their subcellular locations. Immunofluorescence microscopy revealed that both TgSCP and TgAMN were localized to the VAC/ELC **(Fig 5D)**. TgASP1 subcellular location was also determined to be within the VAC (data were deposited in a public repository, www.toxodb.org; Dou, Z. *et al*., in preparation). Collectively, these data suggest that the swollen VAC in Δ*crt* parasites causes reduced transcription and translation of several endolysosomal proteases.

### Suppression of proteolysis within the swollen Δ*crt* VAC partially restores VAC size, organellar separation, and invasion

**Fig 6:**
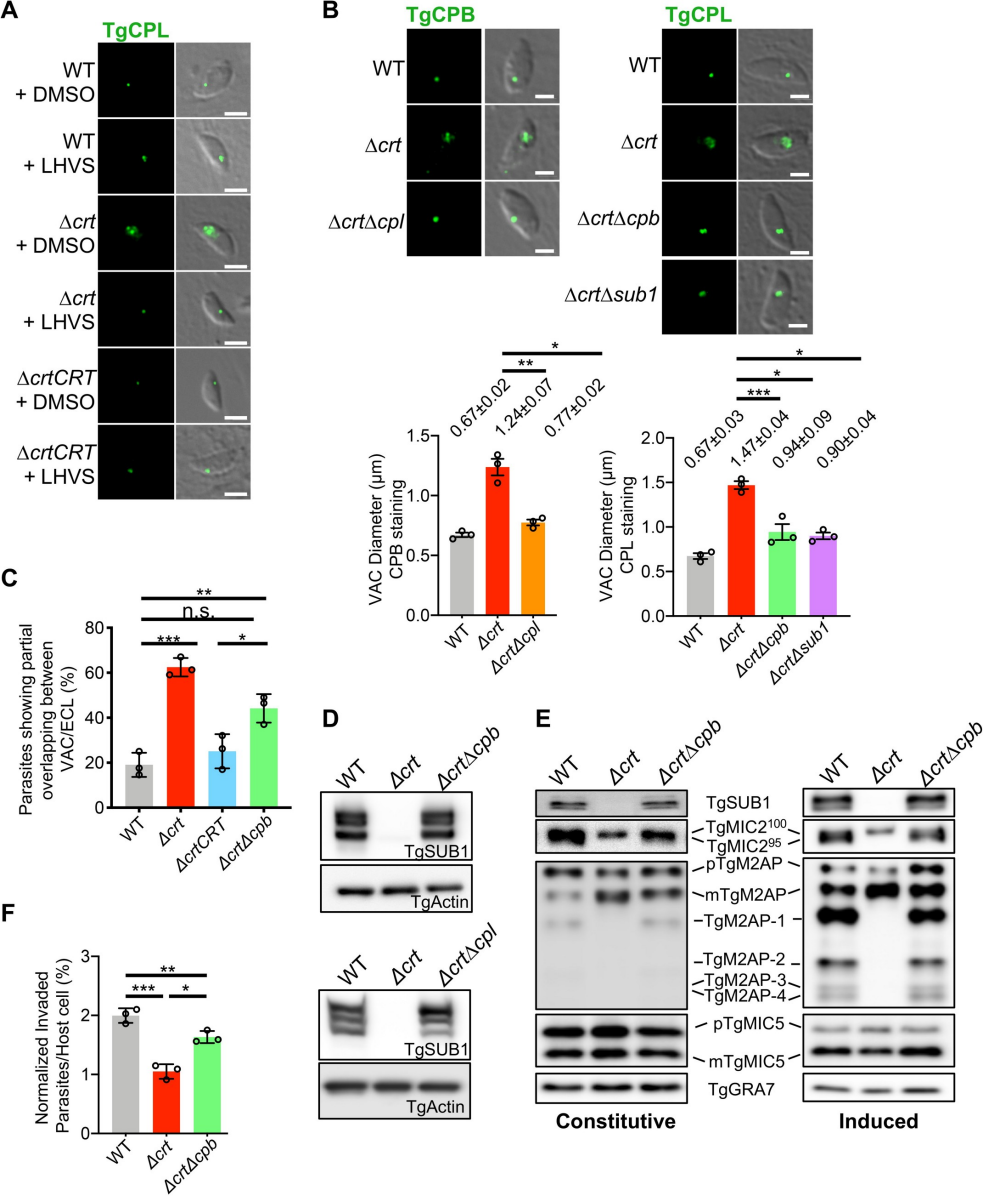
Reduction of proteolytic activity within the swollen Δ*crt* VAC reduced its size and partially restored integrity of the parasite’s endolysosomal system and parasite invasion. (A) The Δ*crt* parasites were incubated with 1 μM LHVS, an irreversible inhibitor of TgCPL, for one lytic cycle, followed by a pulse invasion. Parasites were stained with anti-TgCPL antibodies to determine the size of the VAC. The swollen VAC phenotype was significantly reduced in the LHVS-treated parasites. Scale bar = 2μm. (B) TgCPL and TgCPB, two VAC-residing peptidases, were genetically deleted in the Δ*crt* strain individually to create Δ*crt*Δ*cpl* and Δ*crt*Δ*cpb* double knockouts, respectively. Both Δ*crt*Δ*cpl* and Δ*crt*Δ*cpb* parasites displayed a partial reduction in the size of the VAC compared to WT parasites. Additionally, we genetically deleted *TgSUB1* within the Δ*crt* mutant to create the Δ*crt*Δ*sub1* double knockout. Similarly, the VAC size was partially recovered in the Δ*crt*Δ*sub1* mutant. The VAC sizes are listed in the figure as mean ± SEM. The VAC size measurement was repeated in triplicate. Scale bar = 2 μm. (C) The pulse-invaded parasites showing colocalization between TgCPL (VAC marker) and proTgM2AP (ELC marker) in WT, Δ*crt*, Δ*crtCRT*, and Δ*crt*Δ*cpb* strains were quantified. At least 100 parasites were quantified for each replicate in a total of three replicates. The Δ*crt*Δ*cpb* parasites had a significantly lower percentage of parasites having arrested colocalization between the TgCPL and proTgM2AP compared to the Δ*crt* mutant. (D) The lysates of WT, Δ*crt*, and Δ*crt*Δ*cpl* or Δ*crt*Δ*cpb* parasites were probed with antibodies recognizing TgSUB1. The steady expression of TgSUB1 was restored in both Δ*crt*Δ*cpl* and Δ*crt*Δ*cpb* parasites. (E) The constitutive and induced ESAs of WT, Δ*crt*, and Δ*crt*Δ*cpb* strains were collected and probed with the antibodies indicated in the figure. The secretion and processing of microneme proteins were also recovered in the Δ*crt*Δ*cpb* mutant. (F) The invasion efficiency of WT, Δ*crt*, and Δ*crt*Δ*cpb* strains was determined using the procedures mentioned above. The Δ*crt*Δ*cpb* parasites showed increased invasion efficiency (1.63 ± 0.10 parasites per host cell) compared to the Δ*crt* strain (1.05 ± 0.12 parasites per host cell), albeit still a lower efficiency than the WT parasites (2.00 ± 0.12 parasites per host cell). Statistical significance in all assays listed in this figure was calculated using unpaired two-tailed Student’s *t-*test. *, *p*<0.05; **, *p*<0.01; ***, *p*<0.001; n.s., not significant.

Given that CRT is a putative small solute transporter, the deletion of *TgCRT* potentially results in the accumulation of small nutrient molecules generated by proteolysis within the VAC, further swelling its size. Therefore, we speculated that inhibition of proteolytic activity within the swollen VAC might reduce its size. We tested this hypothesis by chemically or genetically suppressing VAC proteolysis. First, we treated WT, Δ*crt*, and Δ*crtCRT* parasites with 1 μM LHVS, an irreversible inhibitor of TgCPL protease [25]. TgCPL is a major endopeptidase involved in the maturation of microneme proteins and digestion of host proteins [4,11]. Infected host cells were incubated with LHVS for 48 h to allow full inhibition of TgCPL. Treated parasites were liberated from host cells and used to infect new host cells for 30 min, followed by TgCPL staining to quantify the size of the VAC. As expected, LHVS-treated Δ*crt* parasites displayed smaller VACs than DMSO-treated Δ*crt* parasites **(Fig 6A)**. To validate these findings, we genetically ablated *TgCPL* in Δ*crt* parasites to create the Δ*crt*Δ*cpl* double knockout by CRISPR-Cas9-based genome editing. The replacement of *TgCPL* with a pyrimethamine resistance cassette was confirmed by PCR and immunoblotting **(S6A Fig and Table 1)**. The Δ*crt*Δ*cpl* mutant showed a significantly smaller concave structure than Δ*crt* parasites under DIC microscopy **(S7 Fig)**. We also compared the size of the VAC in WT, Δ*crt*, and Δ*crt*Δ*cpl* based on TgCPB staining by immunofluorescence microscopy using similar methods previously described, and found that similarly, the Δ*crt*Δ*cpl* partially reversed its VAC size compared to Δ*crt* parasites, but still showed a larger VAC than the WT strain (**Fig 6B**). Moreover, we deleted *TgCPB* in Δ*crt* to test whether such phenotype of partial VAC size restoration was independent of the deletion of specific VAC/ELC-localizing proteases. TgCPB was previously identified as another known VAC-localizing protease, displaying both endo- and exo-peptidase activities [14,25]. Due to its carboxypeptidase activity, it is expected that TgCPB generates more small solutes relative to TgCPL. We used CRISPR-Cas9 genome editing to generate a Δ*crt*Δ*cpb* double knockout **(S6B Fig and Table 1)**. The successful gene ablation was confirmed by PCR and immunoblotting **(S6B Fig)**. The resulting Δ*crt*Δ*cpb* mutant also showed a smaller concave subcellular structure compared to the Δ*crt* mutant **(S7 Fig)**. The size of the VAC in WT, Δ*crt*, and Δ*crt*Δ*cpb* was quantified based on the TgCPL staining as described above and the Δ*crt*Δ*cpb* parasite VAC was reduced by ~35% compared to Δ*crt* parasites **(Fig 6B)**.

To test if, like TgCPL and TgCPB, residual expression of TgSUB1 in Δ*crt* parasites contributed to VAC swelling, we deleted *TgSUB1* in Δ*crt* to create and validate a Δ*crt*Δ*sub1* double knockout mutant **(S6C Fig and Table 1)**. We found that targeted deletion of *TgSUB1* in Δ*crt* partially reversed the swollen VAC phenotype **(Fig 6B and S7 Fig)**. Altogether, our findings from the chemical or genetic inhibition of different proteases strongly suggest that the proteolysis within the VAC plays a key role in its swelling in parasites lacking *TgCRT*.

Next, we tested whether the partial restoration of VAC size was also associated with the reversal of other phenotypes observed in the Δ*crt* mutant. Here, we chose the Δ*crt*Δ*cpb* strain as a representative strain for further testing. Δ*crt*Δ*cpb* showed fewer parasites with partial overlap between the markers for the VAC (TgCPL) and ELC (proTgM2AP) compared to Δ*crt*. Approximately 44% of Δ*crtΔcpb* parasites showed partial overlap between TgCPL and proTgM2AP staining compared to 62% in the Δ*crt* strain **(Fig 6C)**, with both significantly higher than the 19% and 25% seen for WT and Δ*crtCRT* strains, respectively. We also tested whether the partial resegregation of the VAC and ELC in both Δ*crt*Δ*cpb* and Δ*crt*Δ*cpl* mutants was associated with the recovery of TgSUB1 expression. By immunoblotting, TgSUB1 showed comparable expression in both the WT and Δ*crt*Δ*cpb* strains **(Fig 6D)**. Similarly, such recovery of TgSUB1 expression was also seen in the Δ*crt*Δ*cpl* strain **(Fig 6D)**. Moreover, TgSUB1 secretion was observed in both constitutive and induced ESA fractions in the Δ*crt*Δ*cpb* parasites **(Fig 6E)**. TgM2AP and TgMIC2 were correctly cleaved by TgSUB1 in Δ*crt*Δ*cpb*, and their secretion patterns were similar to those seen in the WT strain **(Fig 6E)**. This partial restoration of phenotypes in the Δ*crt*Δ*cpb* mutant resulted in an increase of invasion efficiency up to ~60% compared to the Δ*crt* strain, although invasion was still significantly lower than that of WT parasites **(Fig 6F)**. Collectively, these data show a close association between the size of the VAC, altered morphology of the parasite’s endolysosomal system, protein abundance of TgSUB1, microneme protein secretion and processing, and parasite invasion.

### TgCRT is a functional transporter

**Fig 7:**
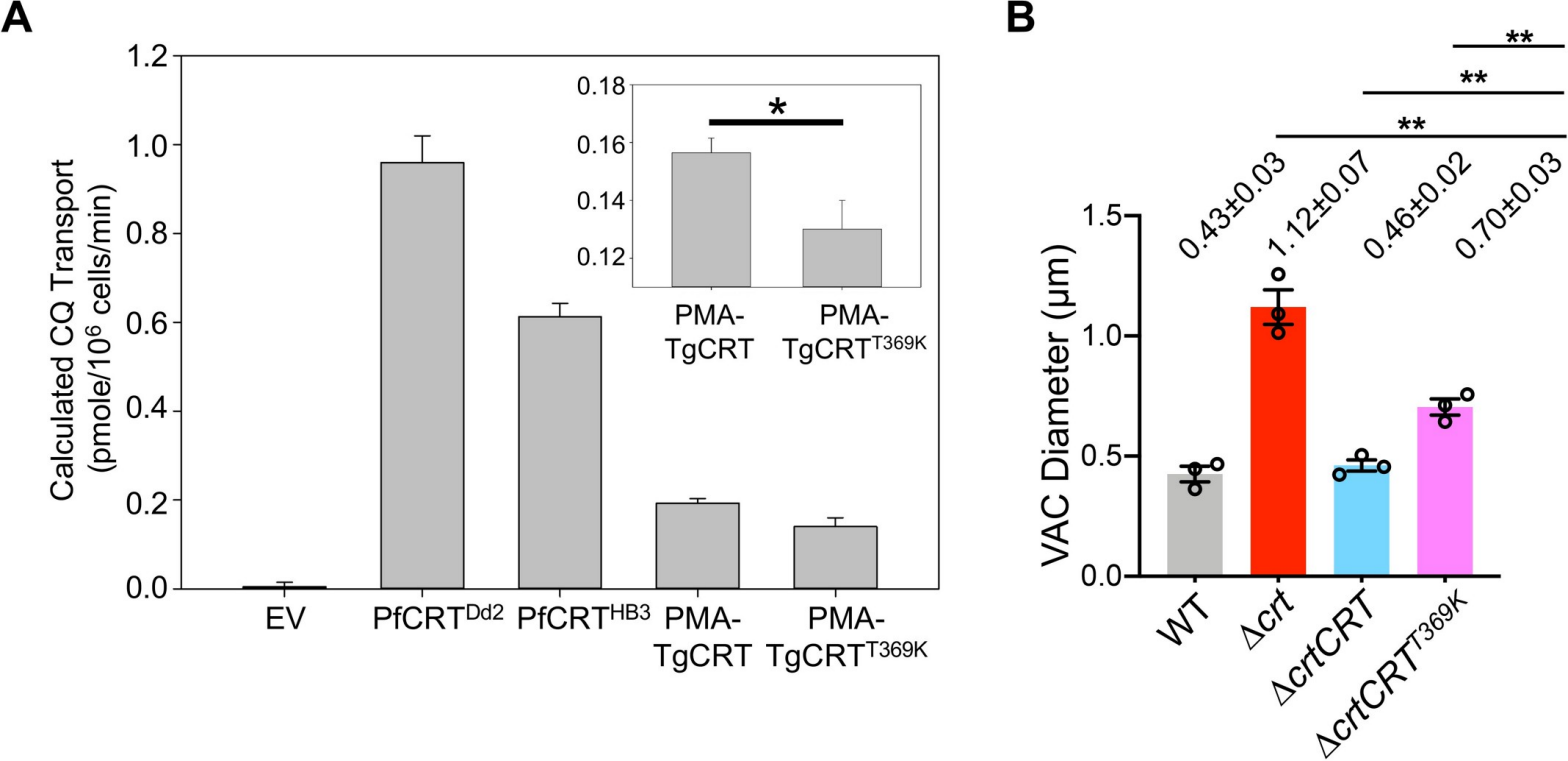
TgCRT is a functional transporter and its transport efficiency is correlated with VAC size in the parasites. (A) CQ transport by PfCRTs and TgCRT (PMA-TgCRT) expressed in *S*. *cerevisiae*. Transport was extrapolated from CQ-induced growth delays as described in [37,39]. Results are the average of at least four independent experiments ± SEM. EV, empty vector; PfCRT^Dd2^, a PfCRT expression construct derived from CQ-resistant malaria strain; PfCRT^HB3^, a PfCRT expression construct derived from CQ-sensitive malaria strain; PMA-TgCRT, plasma membrane ATPase-TgCRT fusion (see S9 Fig); PMA-TgCRT^T369K^, TgCRT fusion protein harboring T to K substitution at the position analogous to residue 76 in PfCRT (see text). (B) A mutation of T369K was introduced into WT TgCRT complementation construct by site-directed mutagenesis before it was electroporated into the Δ*crt* mutant. The VAC sizes were determined based on TgCPL staining using the methods mentioned above. The Δ*crt* mutant complemented with TgCRT^T369K^ partially restored its VAC size, but it was still significantly bigger than that transfected with WT TgCRT. Statistical significance was calculated using unpaired two-tailed Student’s *t-*test. *, *p*<0.05; **, *p*<0.01.

Finally, we attempted to express TgCRT in *S*. *cerevisiae* yeast following previously described strategies for PfCRT [36,37]. Native *TgCRT* cDNA was not expressed well in *S*. *cerevisiae* yeast **(S8 Fig)**. Via alignment with PfCRT **(S9A Fig)**, removing the 300 N-terminal residues of the *TgCRT* gene that are non-homologous to PfCRT preserves all putative transmembraneous domains and inter-helical loop regions. Thus, following a previously published strategy for difficult to express PfCRT mutants [22] we created a fusion gene that replaced the 300 most N-terminal residues of the *TgCRT* sequence with the 111 most N-terminal residues from the *S*. *cerevisiae* plasma membrane ATPase (PMA), which harbors a yeast plasma membrane localization sequence **(S9B and S9C Fig)**. The fusion protein was well expressed in the plasma membrane of *S*. *cerevisiae* **(S8 Fig)** with vacuolar-to-cytosol topology preserved, similar to the expression of PMA-PfCRT in yeast as described in a previous publication [38]. Following an approach previously described for PfCRT and PfCRT mutants [22,39], we assayed PMA-TgCRT expressing yeast for chloroquine (CQ) transport **(Fig 7A).** Via alignment with PfCRT **(S9A Fig)**, TgCRT T369 corresponds to the well-studied K76 residue within PfCRT; previously, mutation of PfCRT K76 to T has been shown to increase the efficiency of CQ transport by PfCRT, although it is not the sole determining factor [22,40,41]. We expressed both WT TgCRT and T369K TgCRT in yeast to measure their transport efficiencies. Both the wild type TgCRT protein and a TgCRT T369K mutant were found to transport CQ, albeit slower than PfCRT^HB3^ and PfCRT^Dd2^ derived from CQ-sensitive and resistant malaria strains, respectively, under similar conditions (**Fig 7A**). Also, we noted that TgCRT required higher external [CQ] (80 mM versus 16 mM for PfCRT) to achieve similar levels of transport, suggesting increased CQ K_m_ for this transporter **(Fig 7A)**. Analysis of PfCRT isoforms and the role that individual amino acid substitutions play in modifying transport activity has been ongoing for well over a decade (*e*.*g*. Callaghan *et al*. 2015 [22]). Since PfCRT—TgCRT identity is modest (**S9A Fig**) many more TgCRT mutagenesis studies will be required to fully define catalytically critical sites in TgCRT. Regardless, these initial data show that mutation of a TgCRT threonine, analogous to the well-known position 76 T for PfCRT [22,40,41], also affects CQ transport as is the case for PfCRT.

We also exchanged threonine for lysine in the WT TgCRT complementation construct and transfected Δ*crt* to examine the extent to which TgCRT^T369K^ affects VAC size in *Toxoplasma* parasites. Interestingly, in contrast to full recovery of VAC size in the WT TgCRT complementation strain, TgCRT^T369K^ only partially restored the swollen VAC **(Fig 7B)**. These findings, along with the TgCRT transport data, strongly suggest that the swollen VAC is caused by luminal osmolyte excess, similar to findings for PfCRT as described in "Discussion".

In summary, our findings strongly suggest a role for TgCRT in small solute transport that impacts VAC volume in the absence of drug transport, similar to the role proposed for PfCRT [18,19]. The identity of the relevant osmoregulatory transport substrate(s) remains to be determined. However, at least for *T*. *gondii*, TgCRT expression is also required for proper segregation of other organelles within the endolysosomal system that, in turn, indirectly facilitates microneme secretion and parasite invasion. The data also indicate that the invasion deficiency exhibited by the Δ*crt* mutant is likely due to multiple factors, since the recovery of TgSUB1 expression and trimming of microneme proteins in Δ*crt*Δ*cpb* did not completely reverse the invasion defects. To the best of our knowledge, this is the first observation of the regulation of the endolysosomal protease expression in apicomplexan parasite by a CRT protein.

## Discussion

*Toxoplasma* utilizes an endolysosomal system to secrete invasion effectors that disseminate infection. These invasion effectors undergo a series of intracellular proteolytic cleavage and trimming steps to reach their final forms. Therefore, maintenance of the integrity of the endolysosomal system is critical for controlling the secretion of invasion effectors in *Toxoplasma*. The Vacuolar Compartment (VAC) (also Plant-Like Vacuole or PLV) is an acidic lysosome-like vacuole. Previous work showed that deletion of a cathepsin L-like protease, a major VAC luminal endopeptidase, leads to invasion, replication, and virulence defects [4,11]. Compromised proteolytic activities within these parasites also result in the inefficient degradation of endocytosed host proteins [11]. Liu *et al*. genetically deleted *TgVP1* in *T*. *gondii* and observed defective secretion and trafficking of microneme proteins, and reduced invasion and virulence in the mutant [16]. Warring *et al*. previously reported that a *Toxoplasma* ortholog of chloroquine resistance transporter (TgCRT) resides in the VAC and that decreased expression of TgCRT leads to swelling of the VAC [15]. However, incomplete reduction of TgCRT expression and lack of systematic dissection of phenotypes in the *TgCRT* knockdown mutant limit understanding of the molecular mechanism by which a dysfunctional VAC affects the parasite.

**Fig 8:**
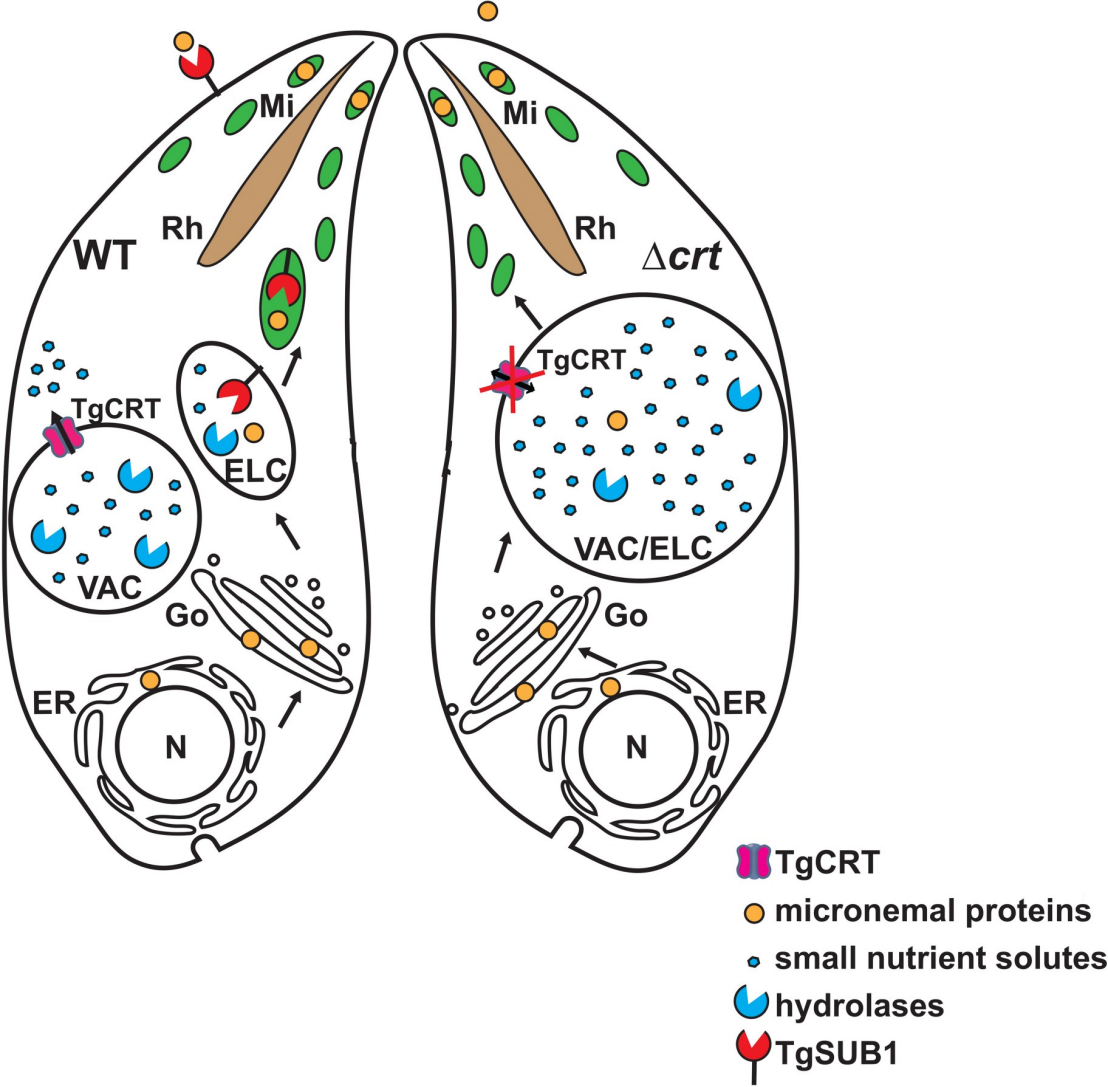
A model for the regulation of the endolysosomal system in *Toxoplasma* parasites. The *Toxoplasma* parasite contains a separate endosome-like compartment from the VAC/PLV within its endolysosomal system. When the parasite lacks TgCRT, the VAC becomes swollen and cannot separate from its adjacent ELC. This aberrant colocalization leads to reduced transcript and protein abundances of several proteases residing within the endolysosomal system, including TgSUB1. These changes alter the secretion of microneme proteins, thereby resulting in invasion defects in the *TgCRT*-null mutant. ELC, endosome-like compartment; ER, endoplasmic reticulum; Go, Golgi apparatus; Mi, microneme; N, nucleus; Rh, rhoptry; TgCRT, *Toxoplasma* chloroquine resistance transporter ortholog; TgSUB1, *Toxoplasma* subtilisin 1; VAC, vacuolar compartment.

Here, we created a *TgCRT* knockout mutant that completely removes *TgCRT* from the VAC membrane. The resulting Δ*crt* strain displays a dramatic increase in VAC size, and the organelle aberrantly overlaps with the adjacent endosome-like compartment **(Fig 8)**. Although a previous study reported that parasites deliver minor amounts of TgCPL to the ELC, which then contributes to maturation of some microneme proteins [4], our data do not reveal abnormal intracellular cleavage or trafficking of microneme proteins in Δ*crt* parasites. This is likely due to the modest decrease of TgCPL expression in Δ*crt*. Relatedly, Dogga *et al*. recently documented that aspartic acid protease 3 (TgASP3) localizes in a post-Golgi compartment and serves as a major maturase for invasion effectors [5].

We also evaluated the processing of microneme proteins by TgROM4 and TgSUB1 in the Δ*crt* mutant. We found that the microneme proteins were improperly trimmed on the surface of Δ*crt* parasites by TgSUB1. Patterns of secreted microneme proteins observed for the Δ*crt* mutant were similar to those for Δ*sub1* parasites, which led us to examine the expression of TgSUB1 in Δ*crt* parasites and ESAs. As expected, levels of TgSUB1 were decreased on the surface of Δ*crt* parasites and in the medium during secretion. Interestingly, the steady-state abundance of TgSUB1 was also significantly decreased in the Δ*crt* mutant. Surprisingly, we found that the reduction of TgSUB1 was due to a decrease in the transcription level of TgSUB1 in the Δ*crt* strain, suggesting that the parasites utilize a transcriptional feedback mechanism to regulate TgSUB1.

TgSUB1 is a micronemal GPI-anchored protein. It remains unclear how TgSUB1 becomes activated within *Toxoplasma*. Previous pulse-chase experiments have revealed that TgSUB1 undergoes maturation in a post-ER compartment, and passes through the endolysosomal system before its arrival at the microneme and subsequent secretion [12]. The propeptide region of TgSUB1 carries targeting information which helps to guide the protein to the micronemes [35]. The propeptide may also function by binding to active sites of mature TgSUB1 to inhibit its proteolytic activity during trafficking. Overlap of the VAC and ELC could bring propeptide-bound TgSUB1 to a protease-abundant environment, where non-specific digestion of the propeptide could then lead to increased digestive activities in the VAC and ultimately result in an increase in osmotic pressure within the hybrid VAC/ELC organelle **(Fig 8)**. In this scenario, the parasites may utilize a feedback mechanism to repress additional expression of TgSUB1 to avoid further VAC swelling. Moreover, we also discovered that the Δ*crt* parasites had reduced protein and/or transcript levels of several other proteases, including two known VAC proteases, TgCPL and TgCPB. Therefore, the parasites down-regulate a number of endolysosomal-VAC proteases to suppress proteolytic activities in the swollen VAC, presumably to reduce osmotic pressure and thereby control VAC size. Among these proteases, TgSUB1 has been shown to be involved in parasite invasion and virulence defects, but not in replication and egress [3]. Additionally, TgCPL plays a role in parasite invasion by contributing to the maturation of at least two microneme proteins [4]. Therefore, the invasion defects exhibited in the Δ*crt* mutant could be due to several factors. Given that the Δ*vp1* mutant showed similar phenotypes as our Δ*crt* mutant, such as reduced secretion of microneme proteins and decreased invasion, we tested whether the loss of TgVP1 can cause swelling of the VAC. However, *TgVP1*-deficient parasites showed normal VAC size and TgSUB1 expression **(S10 Fig)**. Also, higher baseline cytosolic calcium and pH levels previously observed in Δ*vp1* parasites were not seen in Δ*crt* **(S3C and S3D Fig)**. These data suggest that the invasion phenotypes observed within Δ*crt* and Δ*vp1* mutants have different underlying molecular mechanisms.

Altered endolysosomal protease transcript levels in Δ*crt* parasites suggest that parasites repress transcription factors or enhance transcription repressors to respond to increased VAC size. RNA-Seq analysis did not reveal any significant changes in the AP2-family of transcription factors **(S3 Table)**. In mammalian cells, the transcription factor EB (TFEB) is a master regulator that drives gene expression for autophagy and lysosome biogenesis [42]. A search of the *Toxoplasma* genome did not identify a TgTFEB ortholog, suggesting that these parasites may adopt an alternative strategy for regulating lysosomal gene expression. Interestingly, our differential gene expression analysis found that the transcript levels of two zinc finger (CCCH) type motif-containing proteins, TGGT1_246200 and TGGT1_226310, were increased and decreased by 2-fold and 3-fold (**S1 Table**), respectively, in the Δ*crt* mutant. The CCCH type zinc finger motif-containing protein is known to regulate the stability of mRNA [43]. For example, tristetraprolin inhibits the production of tumor necrosis factor-α in macrophages by destabilizing its mRNA via an interaction with AU-rich elements at the 3’-untranslated region [44]. Further investigation to identify transcription factor(s) and regulator(s) that govern the expression of *Toxoplasma* lysosomal genes will help elucidate how these parasites regulate the biogenesis and function of the VAC.

In this study, we have determined that *TgCRT*-deficient parasites have reduced expression of several endolysosomal proteases. We have also found that suppression of proteolytic activities within the swollen VAC decreases the size of the organelle. These findings, along with data verifying that TgCRT is indeed a transporter with function similar to that of PfCRT, support the idea that TgCRT functions to transport essential VAC osmolytes, similar to proposals for PfCRT [18,19,45]. Likely candidate osmolytes include ions and/or amino acids. We suggest that when TgCRT is absent on the membrane of the VAC, protein degradation products (short peptides and amino acids) accumulate within the VAC and increase osmotic pressure, thereby leading to the swollen phenotype. Consistent with this idea, and similar to related observations for *P*. *falciparum* treated with cysteine protease inhibitors [46], chemical inhibition of proteolysis via the small inhibitor LHVS dramatically reduces the size of the VAC. For *Toxoplasma*, LHVS principally targets TgCPL, but also inhibits TgCPB [14]. Therefore, LHVS treatment blocks both of these VAC proteases. Genetic ablation of *TgCPL* or *TgCPB* in Δ*crt* individually resulted in the partial restoration of VAC size. Interestingly, the VAC in Δ*crt*Δ*cpl* parasites was reduced to a greater extent than that in Δ*crt*Δ*cpb*, suggesting that the deletion of *TgCPL* reduces proteolytic activity to a higher extent within the VAC than with the loss of TgCPB alone, which is consistent with a previous finding that the maturation of TgCPB is dependent upon the presence of TgCPL [14]. Additionally, the deletion of *TgCPB* partially restored secretion patterns of microneme proteins and invasion defects. These results also reveal for the first time that TgCPB plays an active role in contributing to proteolysis within the VAC in *Toxoplasma* parasites.

RNA-Seq analysis identified several other genes with altered transcription levels, suggesting that the parasites may utilize additional strategies to control VAC size. For example, interestingly, levels of aquaporin (TGGT1_215450) transcript were reduced in Δ*crt* parasites. Previous work showed that this aquaporin is localized to the VAC or PLV [10]. Therefore, it seems likely that Δ*crt* parasites express less aquaporin to reduce water transport into the VAC/PLV, as an additional tactic to limit VAC swelling. We also found that two putative protein phosphatase 2C (TGGT1_276920 and TGGT1_201520) transcripts are down-regulated in the Δ*crt* mutant, both of which carry signal peptides indicating endosomal trafficking. TGGT1_276920 and TGGT1_201520 are homologous to PTC3 and PTC1 in *S*. *cerevisiae*, respectively. Interestingly, both PTC1 and PTC3 proteins are involved in yeast osmosensory regulation. A mitogen-activated protein kinase pathway is activated when yeast cells experience hyperosmotic conditions. PTC1 and PTC3 negatively regulate this pathway [47,48]. Furthermore, PTC1 was found to control the function and morphology of the yeast vacuole, which further alters its biogenesis [49]. The dramatic change in *Toxoplasma* VAC volume indicates induced osmotic stress in the Δ*crt* parasites. The knockout parasites appear to be utilizing a similar mechanism to suppress these protein phosphatases and enhance similar osmoregulatory signaling. We think that similar studies for *P*. *falciparum* and other apicomplexan parasites that express CRT orthologs would be informative.

In this study, we also determined the acute virulence of *TgCRT*-deficient parasites in a murine model. The Δ*crt* strain did not lead to complete mortality in mice by both subcutaneous and intravenous inoculations. Given the hyper-virulent nature of Type I *Toxoplasma* strain in a murine model, the Δ*crt* mutant dramatically lost its acute virulence compared to WT parasites. We also observed that the Δ*crtCRT* complement did not result in complete mortality when it was used to infect mice by intravenous inoculation. During the natural route of infection, the parasite infection is spread via the host’s circulation system. Therefore, the intravenous inoculation is a more direct route to measure virulence differences in mice than the subcutaneous inoculation, since it circumvents steps of parasite migration from the infection site to the circulation system. Thus, it is possible that our Δ*crtCRT* strain did not fully reverse the acute virulence, albeit significantly recovered the virulence defects compared to Δ*crt* parasites.

The phenotype of the swollen VAC in the Δ*crt* strain mirrors the enlarged digestive vacuole in chloroquine (CQ) resistant (CQR) *P*. *falciparum* malaria [18]. Peptidomic analysis showed that hemoglobin is not as efficiently degraded within the digestive vacuole (DV) in CQR malaria parasites [45], further suggesting that CQR mutations in PfCRT alter the physiology within the swollen digestive vacuole, thereby compromising DV proteolytic activities. *In vitro* assays utilizing recombinant PfCRT, reconstituted in proteoliposomes, have revealed that PfCRT may act as a proton gradient dependent, polyspecific nutrient exporter for small solutes including amino acids, oligopeptides, glutathione, and small drugs [21]. These studies also demonstrate that CQR-associated PfCRTs display altered transport efficiency relative to CQ-associated PfCRT. Our study has revealed that TgCRT mediates CQ transport similar to PfCRT. The Δ*crt* strain appeared more sensitive to CQ relative to WT parasites (**S11 Fig**), further suggesting that TgCRT is a functional transporter of small solutes across the membrane of the VAC. We suggest that alteration of proteolytic activities in the enlarged VAC of the Δ*crt* mutant reveals a similar scenario relative to the CQR *P*. *falciparum* DV. Given the similarity in components and functionality of the VAC and DV found in *Toxoplasma* and *Plasmodium*, this *Toxoplasma TgCRT*-deficient mutant should prove useful for further studies of the native function of CRT orthologs found within other apicomplexan parasites.

In summary, our findings reveal that the *Toxoplasma* TgCRT protein is indeed a small molecule transporter that is required for maintaining the normal size and morphology of the VAC/PLV. Unexpectedly, aberrant swelling of the VAC in *TgCRT*-deficient parasites is also associated with decreased integrity of the parasite’s endolysosomal system, which serves as a conduit for trafficking of invasion effectors. Overlap of the VAC and endosome-like compartment in the *TgCRT* knockout is also associated with a reduction in transcript and protein levels for several endolysosomal proteases. We found that blocking normal proteolysis within the swollen VAC reduced the size and partially restored the morphology of the organelle. Taken together, these findings suggest that TgCRT mediates the transport of small solutes and that putative accumulation of its substrates increases VAC size. The data show that the integrity of the parasite endolysosomal system is necessary for normal parasite virulence. We suggest that pharmaceutical modulation of the VAC could serve as a novel strategy for managing toxoplasmosis.

## Materials and methods

### Ethical statement

This study was performed in compliance with the Public Health Service Policy on Humane Care and Use of Laboratory Animals and Association for the Assessment and Accreditation of Laboratory Animal Care guidelines. The animal protocol was approved by Clemson University’s Institutional Animal Care and Use Committee (Animal Welfare Assurance A3737-01, protocol number AUP2016-012). All efforts were made to minimize discomfort. CO_2_ overdose was used to euthanize mouse. This method of euthanasia is consistent with the recommendations of the Panel on Euthanasia of the American Veterinary Medical Association.

### Chemicals and reagents

Morpholine urea-leucyl-homophenyl-vinyl sulfone phenyl (LHVS) was kindly provided by the Bogyo lab at Stanford University. Fluo-4-AM was purchased from Invitrogen (Life Technologies). Ionomycin was purchased from Sigma Aldrich. All oligonucleotide primers used in this study were listed in S2 Table. Other chemicals used in this work were analytical grade and were purchased from VWR unless otherwise indicated.

### Parasite culture

*Toxoplasma gondii* parasites were cultured in human foreskin fibroblast (HFF) cells (ATCC, SCRC-1041) or hTERT cells in Dulbecco’s Modified Eagle Medium (DMEM) media supplemented with 10% cosmic calf serum at 37°C with 5% CO_2_. The parasites were harvested by membrane filtration as described previously [14].

### Generation of transgenic parasites

To generate the *TgCRT*-deficient strain, ~1.5 kilobases (kb) of the 5’- and 3’-UTR of the *TgCRT* gene, respectively, were PCR-amplified and flanked at both ends of the bleomycin resistance cassette (BLE) to assemble a *TgCRT* deletion construct. The resulting plasmids were introduced into WT parasites by electroporation. The transfected parasites were selected with 50 μg/ml bleomycin twice, while in their extracellular stage as described previously [14]. Clones of the *TgCRT*-deficient parasites were isolated by limiting dilution. The correct replacement of *TgCRT* with the *BLE* cassette was confirmed by PCR (see S1 Text).

To complement Δ*crt* parasites, we modified the plasmid pTub-TgCRT-mCherry-3xmyc (a gift from the van Dooren lab), which expresses a C-terminally mCherry-3xmyc epitope-tagged TgCRT under the *Toxoplasma* tubulin promoter. The plasmid was digested with HpaI and MfeI restriction endonucleases to remove the tubulin promoter and a segment of *TgCRT*. The remaining DNA fragment served as the backbone for subsequent Gibson assembly to incorporate a PCR amplified ~1 kb region upstream of the *Tgku80* gene, the ~1 kb fragment of the *TgCRT* 5’-UTR region, and the removed partial *TgCRT* coding sequence to produce the TgCRT complementation plasmid, pCRT-TgCRT-mCherry-3xmyc. The complemented TgCRT is driven by its cognate promoter to maintain physiologic similarity to native TgCRT expression in WT parasites. The 1 kb region located ~6 kb upstream of the *TgKu80* gene was used to facilitate a single integration of the TgCRT complementation plasmid into this specific locus by single crossover homologous recombination. The TgCRT complementation construct was digested with SwaI restriction endonuclease enzyme, gel-extracted, purified, and transfected into Δ*crt* parasites by electroporation.

To introduce NanoLuc luciferase (nLuc) into parasites, we PCR-amplified and assembled the *TgTubulin* promoter, the coding sequence of the nLuc luciferase, and an HXG selection marker into a nLuc expression construct. The resulting plasmid was transfected into WT, Δ*crt*, and Δ*crtCRT* strains. The transfectants were selected with 25 μg/ml mycophenolic acid and 50 μg/ml xanthine. Stable populations were subjected to limiting dilution to generate individual clones of WT::*nLuc*, Δ*crt*::*nLuc*, and Δ*crtCRT*::*nLuc* and clones were confirmed via luciferase activity.

To generate the Δ*crt*Δ*cpb* mutant, the *TgCPB* gene was replaced with a pyrimethamine resistance cassette using the CRISPR-Cas9 genome editing system [50,51]. The pyrimethamine resistance cassette was PCR-amplified and flanked by 50 bp regions upstream and downstream of the start and stop codons of the *TgCPB* gene for homologous recombination. A 20 bp region located at the beginning of the coding region of the *TgCPB* gene was used to design guide RNA and replace the guide RNA targeting *TgUPRT* gene in the plasmid pSAG1-Cas9::UPRTsgRNA using Q5 site-directed mutagenesis (NEB). The Cas9-GFP and guide RNA constructs were co-transfected into Δ*crt* parasites with the corresponding repair PCR product. The guide RNA and Cas9 generated a gap within the *TgCPB* gene to facilitate double crossover homologous recombination. Correct gene replacement was confirmed by PCR. Similar strategies were used to create the Δ*crt*Δ*cpl* and Δ*crt*Δ*sub1* mutants. Please refer to the figure legend of S6 Fig for more details.

To epitope-tag TgAMN, we again used CRISPR-Cas9 editing tools to modify the corresponding gene. Guide RNA recognizing the 20 bp region near the TgAMN stop codon was generated using the methods above. The 50-bp homologous regions upstream and downstream of the stop codon of the *TgAMN* gene were cloned at the 5’- and 3’-ends of the DNA sequence containing the 3xHA epitope tag and the pyrimethamine resistance cassette, respectively, by PCR. The plasmid encoding the guide RNA targeting TgAMN and Cas9-GFP and the PCR product were co-transfected into WT parasites. The stop codon of TgAMN was replaced by the 3xHA epitope tag and pyrimethamine resistance cassette. Stable populations were generated after multiple rounds of pyrimethamine selection and the TgAMN-3xHA fusion protein was confirmed by immunoblotting analysis.

TgSCP was endogenously tagged with a 3xmyc epitope tag via single crossover. An approximately 1 kb region upstream of the *TgSCP* stop codon was PCR amplified and fused in frame with a 3xmyc epitope to assemble TgSCP-3xmyc. A pyrimethamine resistance cassette was also included, the resulting plasmid was linearized and transfected into WT parasites. The correct tagging was confirmed by immunoblotting.

### Site-directed mutagenesis

Threonine 369 was mutated to lysine in the WT TgCRT complementation construct, via site-directed mutagenesis according to the Q5 site-directed mutagenesis procedure (NEB). Linear PCR product was phosphorylated, circularized, and transformed into *E*. *coli*. Correct clones were identified by direct DNA sequencing.

### Transfection of *Toxoplasma* parasites

*T*. *gondii* parasites were allowed to grow in HFF cells for 48 h at 37°C with 5% CO_2_. Freshly egressed parasites were syringed, filter purified, and harvested in Cytomix buffer (25 mM HEPES, pH 7.6, 120 mM KCl, 10 mM K_2_HPO4/ KH_2_PO4, 5 mM MgCl_2_. 0.15 mM CaCl_2_, and 2 mM EGTA). Parasites were pelleted at 1,000x *g* for 10 min, washed once in Cytomix buffer, and resuspended in Cytomix buffer at 2.5 x 10^7^ parasites per ml. 400 μl of parasite suspension was mixed with 20 μg DNA and 2 mM ATP/5 mM reduced glutathione to a final volume of 500 μl. The mixture was electroporated at 2 kV and 50 ohm resistance using the BTX Gemini X2 (Harvard Apparatus). Transfectants were inoculated into a T25 flask pre-seeded with a confluent monolayer of HFF cells. The transfected parasites were allowed to recover prior to drug selection applied 24 h post transfection.

### Immunofluorescence and colocalization analysis

Freshly lysed parasites were used to infect confluent HFF cells pre-seeded in an 8-well chamber slide for 1 hr (pulse-invaded parasites) or 18–24 h (replicated parasites). The extracellular parasites were attached to chamber slides using 0.1% (w/v) poly-L-lysine. Immunofluorescence was performed as described previously [11,14]. Images were viewed and digitally captured using a Leica CCD camera equipped with a DMi8 inverted epifluorescence microscope and processed with Leica LAS X software. For deconvolution microscopy, a series of Z-stack images were captured and processed by the 3-D deconvolution operation embedded in the Leica LAS X software using the following parameters: Total iterations: 10; Refractive index: 1.52; Method: Blind; Remove background: Yes; Rescale intensity: Yes; AutoQuant Deconvolution algorithms licensed from Media Cybernetics Inc. The fluorescence in final images was presented as maximum projection. The colocalization analysis of two proteins of interests was performed by the Coloc2 plugin embedded in the Fuji image processing suite using the following parameters [52]: Threshold regression, Bisection; Algorithms, Li for both channels. Pearson’s correlation coefficient above threshold was recorded and plotted for comparison. The colocalization analysis was derived from 10 parasites per replicate for a total of three replicates. The statistical analysis was calculated by unpaired two-tailed Student’s *t*-test.

### Measurement of cytosolic pH in *Toxoplasma* via pHluorin 2 (PHL2)

To express PHL2 in the cytoplasm of the parasites, the gene encoding PHL2 was cloned under the *Toxoplasma* tubulin promoter in a plasmid carrying a chloramphenicol resistance cassette for selection. The resulting plasmids were introduced into WT, Δ*crt*, and Δ*crtCRT* strains by electroporation. After drug selection, the parasite strains expressing PHL2 were cloned out by limiting dilution. Individual clones were confirmed by fluorescence observation via immunofluorescence microscopy. Prior to pH measurement, a calibration curve was generated by measuring the ratio of pHL2 fluorescence excited at 405 nm and 485 nm in the WT strain expressing cytosolic pHL2 according to previously published methods [53]. Briefly, the PHL2-expressing WT parasites were filter-purified, resuspend in PBS at 1x10^7^ parasites per ml. One hundred microliters of parasites were pelleted and resuspended in buffer with different pH values ranging from 6.2 to 7.8. The detergent, Triton X-100, was added into the parasite resuspensions to 0.1% and incubated at room temperature for 10 min to permeabilize parasite cell membranes for PHL2 release. Released PHL2 was excited at 405 and 485 nm and the emitted fluorescence at 528 nm was recorded via a BioTek Synergy H1 fluorescence plate reader. The ratio of the emission intensities at 528 nm (I405/I485) for both excitation wavelengths were plotted, which yields a calibration equation by linear regression analysis. Cytosolic PHL2 expressing WT, *Δcrt*, and Δ*crtCRT* parasites were harvested, resuspended in PBS, and their I405/I485 ratios were measured. The pH of their cytosol was calculated by applying their I405/I485 ratios to the calibration equation. All assays were repeated in three technical replicates per biological replicate in a total of three biological replicates. Data were presented as mean ± SEM.

### Excretory secretory antigens (ESAs) preparation

Freshly egressed parasites were syringed, filter purified, and resuspended at 5 x 10^8^ parasites/ml in D1 medium (DMEM medium supplemented with 1% FBS). One hundred microliters of parasite suspension were transferred to a microfuge tube and incubated at 37°C for 30 min to prepare constitutive ESAs. To isolate induced ESAs, the parasite suspension was incubated in D1 medium supplemented with 1% ethanol for 2 min at 37°C. ESAs were separated from intact parasites by centrifugation at 1,000 x *g* for 10 min. ESA fractions were transferred to a new microfuge tube, mixed with SDS-PAGE sample loading buffer, and boiled for 5 min for immunoblotting analysis.

### SDS-PAGE and immunoblotting

Parasite lysates and ESA fractions were prepared in 1x SDS-PAGE sample buffer and boiled for 5 min before resolving on standard SDS-PAGE gels. For immunoblotting, gels were transferred to PVDF membranes by semi-dry protein transfer methods. Blots were blocked with 5% non-fat milk and incubated with primary antibody diluted in 1% non-fat milk. Goat anti-mouse or anti-rabbit IgG antibodies conjugated with horseradish peroxidase were used as the secondary antibody. Immunoblots were developed with SuperSignal WestPico chemiluminescent substrate (Thermo). The chemiluminescence signals were captured using the Azure Imaging System. Bands were quantified by densitometry using LI-COR Image Studio software.

### Parasite invasion and attachment assay

The red-green invasion assay was used to measure the efficiency of parasite invasion. Freshly purified parasites were syringed, filter purified, and resuspended at 5 x 10^7^ parasites/ml in invasion medium (DMEM supplemented with 3% FBS). Two hundred microliters of parasite resuspension were inoculated into each well of an 8-well chamber slide pre-seeded with HFF cells, and parasites were allowed to invade host cells for 30, 60, and 120 min before fixation with 4% formaldehyde for 20 min. Before membrane permeabilization, slides were stained with mouse anti-TgSAG1 monoclonal antibody (1:1,000) for 1 h to label attached parasites. After treatment with 0.1% Triton X-100 for 10 min, the parasites were stained with rabbit polyclonal anti-TgMIC5 antibody (1:1,000) for 1 h to stain both invaded and attached parasites. Subsequently, slides were stained with goat anti-mouse IgG conjugated with Alexa 594 (red) (Invitrogen, 1:1,000) and goat anti-rabbit IgG conjugated with Alexa 488 (green) (Invitrogen, 1:1,000) along with DAPI for nuclear staining. After staining, slides were mounted with anti-fade Mowiol solution and observed by immunofluorescence. Intracellular parasites only showed green fluorescence, whereas extracellular parasites exhibited both red and green fluorescence. Six fields of view from individual invasion experiments were captured by a Leica DMi8 inverted epifluorescence microscope and processed with ImageJ software. The invasion efficiency of each strain was quantified using the following equation ([sum of green parasites] − [sum of red parasites]) ⁄ total host nuclei.

For attachment assay, the HFF cells pre-seeded in 8-well chamber slides were fixed with 2% glutaraldehyde for 10 min at 4°C followed by PBS wash and quench with 1M glycine overnight. The fixed HFF cells were incubated with D3 medium (DMEM supplemented with 3% FBS) for 1 h at 37°C before parasite inoculation. Freshly filter-purified parasites were resuspended at 5x10^7^ parasites per ml in D3 medium (DMEM supplemented with 3% FBS). Two hundred microliters of parasite resuspension (1x10^7^) were added into each well and allowed to attach onto fixed HFF cells for 15 min at 37°C. Unattached parasites were washed off by PBS rinse 10 times. Parasites attached to HFF cells were fixed with 4% paraformaldehyde for 20 min at room temperature and stained with mouse anti-TgSAG1 antibodies for 1 h at room temperature. Goat anti-mouse IgG conjugated with Alexa 594 (red) (Invitrogen, 1:1,000) was used as secondary antibody. Eight view fields in each well for individual strains were captured at 200x magnification and quantified. The assay was repeated in three biological replicates. Final data were combined and plotted using Prism software. The unpaired two-tailed Student’s *t*-test was used to calculate statistical significance.

### Immunofluorescence-based replication assay

Freshly egressed parasites were filter-purified and inoculated into individual wells of an 8-well chamber slide pre-seeded with HFF cells at approximately 1 x 10^5^ cells per well. Non-invaded parasites were washed off at 4 h post-infection. Invaded parasites were allowed to infect host cells for an additional 24 and 36 h before fixation. The infected host cells were stained with monoclonal anti-TgGRA7 (1:1,000) antibody and DAPI to help distinguish individual parasitophorous vacuoles (PVs) and the nuclei of parasites, respectively. Slides were subjected to standard immunofluorescence microscopy for imaging. One hundred parasitophorous vacuoles were enumerated for each strain and plotted as the distribution of different sized PVs. In addition, replication was also expressed as the average number of parasites per PV.

### Luminescence-based growth assay

Parasites expressing NanoLuc luciferase were inoculated into a white 96-well tissue culture plate with a flat, solid bottom (Greiner Bio-One) pre-seeded with confluent HFF cells at 1.5 x 10^3^ cells/well. Each strain was inoculated into 4 individual wells to monitor the fold-change of luciferase activity versus time, which is proportional to intracellular growth. At 4 h post-infection, the individual wells were aspirated to remove non-invaded parasites. The first well was treated with 100 μl of lysis buffer containing NanoLuc luciferase substrate and incubated for 10 min, and a luminescence reading was taken by using the BioTek multimode H1 hybrid plate reader. The remainder of the 3 wells were replenished with fresh D10 medium without phenol red for an additional 24, 48, and 72 h. Subsequent luminescence readings were all performed via the methods above. Luminescence readings versus time were normalized against the reading at 4 h post-infection to calculate the fold-change of parasite growth.

### Egress assay

A lactate dehydrogenase release assay was used to measure the egress efficiency of parasites. Freshly lysed parasites were filter-purified and resuspended in D10 medium at 5 x 10^5^ parasites/ml. One hundred microliters of parasite suspension were inoculated into each well of a 96-well plate pre-seeded with HFF cells. The parasites were allowed to replicate for 18–24 h, washed, and incubated with 50 μl of Ringer’s buffer (10 mM HEPES, pH 7.2, 3 mM HaH_2_PO_4_, 1 mM MgCl_2_, 2 mM CaCl_2_, 3 mM KCl, 115 mM NaCl, 10 mM glucose, and 1% FBS) for 20 min. Subsequently, an equal volume of 1 mM Zaprinast dissolved in Ringer’s buffer was added to the wells and incubated for 5 min at 37°C and 5% CO_2_. Uninfected wells were treated with 50 μl of Ringer’s buffer containing 1% Triton X-100 or normal Ringer’s buffer, serving as positive and negative controls, respectively. The released lactate dehydrogenase was centrifuged at 1,000 *x g* for 5 min twice to pellet insoluble cell debris. Fifty microliters of supernatant were subjected to the standard lactate dehydrogenase release assay as described previously [54]. The egress efficiency of each strain was calculated using the following equation, ([LDH activity derived from individual parasites]− [LDH activity of negative control]) ⁄ ([LDH activity of positive control]−[LDH activity of negative control]).

### Chemically induced motility assay

Twenty-four hours prior to microscopy, 35 mm MatTek dishes (MatTek Corporation) were incubated with 10% fetal bovine serum (FBS) to provide sufficient protein to form a surface conducive to motility. MatTek dishes were washed once with PBS and loaded with 2 ml of Ringer buffer without Ca^2+^. MatTek dishes were chilled on ice and parasites were added and allowed to equilibrate for 15 min. To remove parasites that have not attached, dishes were washed twice with 2 mL of Ringer buffer without Ca^2+^. Dishes were then placed in the General Electric Delta Vision environmental chamber set to 37°C and allowed to equilibrate for 5 min. Time-lapse videos were taken and photographed using an Olympus IX-71 inverted fluorescence microscope with a Photometrix CoolSnapHQ CCD camera driven by Delta Vision software. The exposure duration, gain, laser intensity, and filter settings were identical for all videos taken for quantification. After 30 seconds, 1 μM Ionomycin was added to stimulate motility. Tracings were quantified for two different measurements: A) for circular motility, the total numbers of parasites in the field of view were divided by the total number of parasites completing at least one full circle. Data presented were from 6 independent trials. B) for calculating total distance traveled, ImageJ software with the MTrackJ plugin was used to track and calculate distance. We report the average distance of a minimum of three parasites (out of a maximum of 5) from 5–6 independent biological trials.

### Measurement of cytoplasmic calcium with Fura2

Intracellular calcium was determined fluorometrically by loading parasites with Fura 2-AM as described previously [55]. Briefly, parasites were harvested, washed with buffer A (116 mM NaCl, 5.4 mM KCl, 0.8 mM MgSO_4_, 5.5 mM D-glucose, and 50 mM HEPES, pH 7.4) with glucose (BAG), and filtered through a 5 μm filter. Parasites were resuspended to 1 x 10^9^ parasites/ml in BAG supplemented with 1.5% sucrose and 5 μM Fura 2-AM. Parasite suspensions were incubated for 26 min in a 26°C water bath with mild agitation. Cells were washed twice with BAG to remove extracellular dye and were resuspended to a final density of 1 x 10^9^ cells/ml. A 50 μl aliquot of the cell suspension was added to 2.45 ml of Ringer buffer without calcium in a cuvette placed in an Hitachi F-7000 fluorescence spectrometer (Hitachi). The excitations were set at 340 and 380 nm, and emission at 510 nm. The Fura2 response was calibrated from the ratio of 340/380 nm fluorescence values [55]. To determine the initial calcium levels via the chemical indicator, initial calcium readings from parasites were determined from 4 independent trials by averaging the first 50 seconds of each tracing.

### Size measurement of the VAC

The size of the VAC was quantified based on TgCPL or TgCPB staining (both TgCPL and TgCPB are VAC luminal proteases). Freshly purified parasites were inoculated into pre-seeded HFF chamber slides, allowed to invade host cells for 30 min prior to fixation, stained with polyclonal rabbit anti-TgCPL antibody (1:100) or mouse anti-TgCPB antibody (1:500), and VAC diameter measured by immunofluorescence microscopy. The distance of the widest diagonal of TgCPL or TgCPB staining was used as the diameter of the VAC and was quantified using Leica LAS X software. Measurements for at least 50 individual parasites were performed for each replicate in a total of three replicates. The measurements are presented as mean ± SEM (standard error of mean).

### Transcriptome sequencing and quantitative PCR (qPCR) assay

WT and *TgCRT*-deficient parasites were grown in HFF cells for 2 days. The parasites were syringed, filter-purified, and resuspended in ice-cold PBS buffer. A trizol-based method was used to extracted total RNA by using the Direct-zol RNA MiniPrep Plus kit (Zymo). Purified total RNA was converted to sequencing read libraries using the TruSeq Stranded mRNA sequencing kit (Illumina). The prepared libraries were subjected to 2 x 125 bp paired-end Illumina HiSeq2500 sequencing. Each sample was sequenced to a depth of at least 20 million reads. The sequencing reads per sample were trimmed and mapped to the genome of *Toxoplasma* GT1 strain (release 34) for gene differential expression profiling by the Clemson University Genomics Computational Lab.

Approximately 500 ng of total RNA was used to measure the steady levels of transcripts for individual genes by using the Luna Universal One-Step RT-PCR kit (NEB). The qPCR assay was performed using the BioRad CFX96 Touch Real-Time PCR detection system. The cycle threshold (CT) values for individual genes were used for double delta CT (ΔΔCT) analysis to calculate their relative abundances to that of WT parasites using the Bio-Rad CFX Maestro software. *TgActin* was used as the housekeeping gene for normalization.

### Mouse studies

Six- to eight-week-old, outbred CD-1 mice were infected by subcutaneous or intravenous injection with 100 WT or mutant parasites diluted in PBS. The infected mice were monitored for symptoms daily for a total of 30 days. Mice that appeared moribund were humanely euthanized via CO_2_ overdose, in compliance with Clemson University IACUC’s approved protocol. The seroconversion of the surviving mice was tested by enzyme-linked immunosorbent assay (ELISA). The surviving mice were allowed to rest for 10 days, prior to subcutaneous injection with a challenge dose of 1000 WT parasites, and were monitored daily for survival for additional 30 days.

### Generation of *TgCRT* expression construct in yeast

*TgCRT* cDNA was PCR-amplified from pTub-TgCRT-mCherry-3xmyc plasmid using a forward primer that introduced a 5’ KpnI site and *S*. *cerevisiae* Kozak sequence, and a reverse primer that omitted the mCherry-3xmyc tag and introduced a 3’ XmaJI site. The PCR amplified DNA was digested with KpnI and XmaJI and subcloned into pYES2-6xHis-BAD-V5 (hexa His, biotin acceptor domain, V5 tags) plasmid behind the GAL1 promoter and in front of the His-BAD-V5 epitope tags to generate the plasmid pYES/TgCRT-hbv. To generate the plasmid pYES/PMA-TgCRT-hbv, DNA encoding TgCRT-hbv was PCR amplified using a forward primer that omitted the first 900 bases of TgCRT and introduced a 5’ SacI site, and a reverse primer that included a 3’ NotI site and His-BAD-V5 tags. The amplified DNA was digested with SacI and NotI and subcloned into a SacI/NotI-digested pYES/PfHB3_PMA_ (from [37]; modified via site-directed mutagenesis to introduce a SacI site at the PMA-PfCRT interface). Mutagenesis reactions were performed using reagents obtained from Agilent (Santa Clara, CA).

### Preparation of yeast membrane and immunoblotting

Isolation of yeast membranes and detection of proteins by Western blot were completed using previously published methods [22].

### Measurement of CQ incorporation in yeast expressing CRT

Quantitative growth rate analysis was used to calculate CQ transport as previously described in detail elsewhere [22,37,39]. Briefly, growth under each condition was measured in duplicate at an initial cell density of OD_600_ = 0.1 in 96-well plates placed in a Tecan (Durham, NC) M200Pro or BioTek (Winooski, VT) Epoch2 plate reader. CQ-induced growth delays at 80 mM CQ, pH 6.75 were calculated as the difference in time taken to reach maximal growth rate in PMA-TgCRT non-inducing versus inducing media (see [39]).

### Statistics

Statistical analysis was performed using Prism software (GraphPad version 8). The methods used in different assays were indicated in the figure legends. All statistical significance analysis in this paper used the raw data sets for calculation (refer to S2 Text for raw data sets).

## Supporting information

S1 Fig Deletion of *TgCRT* in *Toxoplasma* parasites and generation of a *TgCRT* complementation strain. (A) Schematic illustration of the strategies for the *TgCRT* deletion and complementation in *Toxoplasma* parasites. The plasmid carrying a bleomycin resistance cassette (*BLE*) flanked by the *TgCRT* targeting sequences was transfected into WT parasites for double-crossover replacement of *TgCRT* to produce the Δ*crt* strain. The TgCRT complementation plasmid, containing the coding sequence of *TgCRT* fused with mCherry and 3xmyc epitope tags at its C-terminus, was introduced into the Δ*crt* strain to produce the Δ*crtCRT* complementation strain. (B) The primers indicated in panel A were used to verify the correct replacement of *TgCRT* with *BLE* by PCR. (C) The complemented *TgCRT* gene was transfected into Δ*crt* parasites and verified by PCR. Since we complemented Δ*crt* with the coding sequence of *TgCRT*, the PCR product was a 0.2 kb fragment in the Δ*crtCRT* strain, whereas showing a 1.5 kb product in the WT strain whose *TgCRT* gene contains the introns. (D) Transcript levels of *TgCRT* in the WT, Δ*crt*, and Δ*crtCRT* strains were evaluated by quantitative PCR. Primers were designed to anneal to the exons of *TgCRT* and are indicated in panel A. The *TgActin* gene was included as a control for normalization. The quantification of transcripts was performed in three biological replicates and analyzed using unpaired two-tailed Student’s *t-*test. **, *p*<0.01; n.s., not significant.
(TIF)

S2 Fig TgCRT did not co-localize with TgNHE3 in the Δ*crt* mutant. Pulse invaded and replicated parasites were co-stained with anti-TgCPL (the marker of the VAC) and anti-TgNHE3 (one marker of the ELC). The TgCRT and TgNHE3 staining in the Δ*crt* parasites at both stages were juxtaposed similarly to that which was seen in the WT and Δ*crtCRT* strains. The scale bars in the images of pulse invaded and replicated parasites are 2 μm and 5 μm, respectively.
(TIF)

S3 Fig Additional phenotypic characterization of Δ*crt* mutant. (A) The extent of the invasion defects in the Δ*crt* mutant was gradually minimized over time. At 60 min post-infection, there was approximately a 20% reduction in invasion in the Δ*crt* mutant (11.45 ± 0.76) compared to WT (14.49 ± 1.68) and Δ*crtCRT* (12.88 ± 3.19) strains. At 120 min post-infection, WT, Δ*crt*, and Δ*crtCRT* strains displayed 37.03 ± 6.42, 45.14 ± 8.14, and 50.02 ± 12.77 parasites per host cell, respectively, which did not indicate significant invasion differences among these three strains at this time point. The assay was performed in triplicate. (B) We compared the gliding distances and types of WT, Δ*crt*, and Δ*crtCRT* strains, and did not observe significant defects in gliding motility for the Δ*crt* mutant. All of the assays were repeated in 5–6 replicates. (C) The baseline cytosolic calcium concentrations among these strains were evaluated by using ratiometric fluorescence measurements. Comparable calcium levels were observed in the cytoplasm of WT, Δ*crt*, and Δ*crtCRT* parasites. Calcium quantification was repeated in 4 replicates. (D) The cytosolic pH was determined by introducing a ratiometric pH-sensitive fluorescent protein, named pHluorin 2 (PHL2) into these strains. The cytosolic pH among these strains was calculated by applying the fluorescence ratio of the PHL2 excited at 405 and 485 nm to an equation deduced from a calibration curve. Three independent replicates were performed. No cytosolic pH differences were observed among these strains. Statistical significance in all assays listed in this figure was determined using unpaired two-tailed Student’s *t*-test. *, *p*<0.05; n.s., not significant.
(TIF)

S4 Fig No defects in intramembrane proteolytic cleavage of microneme protein 2 (TgMIC2) were observed in the Δ*crt* mutant. Purified, extracellular parasites that had not been permeabilized were stained with anti-TgMIC2 and anti-TgSAG1 antibodies in order to measure the retention of TgMIC2 on the parasite surface. During secretion, the TgMIC2 protein is cleaved by intramembrane rhomboid proteases, such as TgROM4. The abundance of TgMIC2 on the surface of Δ*crt* parasites was similar to that of the WT and Δ*crtCRT* strains, indicating that there is comparable intramembrane cleavage of TgMIC2 among the parasites with or without TgCRT.
(TIF)

S5 Fig Schematic of the endogenous epitope-tagging of *Toxoplasma* putative aminopeptidase N (TgAMN, TGGT1_221310) and putative Pro-Xaa serine carboxypeptidase (TgSCP, TGGT1_254010). (A) The plasmids encoding Cas9 and sgRNA targeting TgAMN were co-transfected into WT parasites with the PCR product carrying a 3xHA epitope tag and a pyrimethamine resistance cassette (DHFR) flanked by 50 bp regions upstream and downstream of the stop codon of TgAMN. The 3xHA tag and the drug resistance cassette were incorporated at the C-terminus of the *Toxoplasma* putative aminopeptidase N via double crossover homologous recombination mediated by the CRISPR-Cas9 genome editing tool. (B) The putative Pro-Xaa serine carboxypeptidase was endogenously tagged with a 3xmyc epitope tag at its C-terminus by single crossover homologous recombination. A 1 kb region upstream of the stop codon of TgSCP was amplified and fused at the 5’-end of the 3xmyc tag to produce the TgSCP-3xmyc tagged plasmid. The 1 kb TgSCP-coding region was cleaved by an endonuclease in the middle prior to transfection to facilitate its integration.
(TIF)

S6 Fig Genetic ablation of genes encoding VAC/ELC-localizing proteases in *Toxoplasma* Δ*crt* parasites. (A) Schematic illustration for the generation of the Δ*crt*Δ*cpl* mutant. A PCR product carrying a pyrimethamine resistance cassette (DHFR) flanked by 50 bps of the 5’- and 3’-untranscribed regions of *TgCPL* was transfected into Δ*crt* parasites for double-crossover replacement of *TgCPL*. Primers indicated in panel A were used to verify the replacement of *TgCPL* with *DHFR* via PCR and agarose gel electrophoresis. The ablation of *TgCPL* in Δ*crt*Δ*cpl* parasites was also confirmed by immunoblotting. (B) A similar strategy was used for the generation of the Δ*crt*Δ*cpb* mutant. The mutant was confirmed by PCR and immunoblotting. (C) The *TgSUB1* gene was also genetically deleted from the Δ*crt* mutant by using a similar method. PCR and immunoblotting were used to confirm the deletion of *TgSUB1*. The TgSUB1 protein level was found to be dramatically reduced in Δ*crt*, but a residual amount of TgSUB1 was still observed compared to the Δ*crt*Δ*sub1* mutant.
(TIF)

S7 Fig The subcellular concave structures in the Δ*crt*Δ*cpl*, Δ*crt*Δ*cpb*, *and* Δ*crt*Δ*sub1* mutants were partially shrunken relative to the Δ*crt* strain during the extracellular stage. WT, Δ*crt*, Δ*crtCRT*, Δ*crt*Δ*cpl*, Δ*crt*Δ*cpb*, and Δ*crt*Δ*sub1* parasites were purified and attached to the surface of a slide for differential interference contrast (DIC) microscopy imaging. Although the *crt*Δ*cpl*, Δ*crt*Δ*cpb*, and Δ*crt*Δ*sub1* mutants still showed an enlarged concave subcellular structure (indicated by the arrow), their sizes were significantly smaller than those in the Δ*crt* mutant. Scale bar = 5 μm.
(TIF)

S8 Fig Anti-V5 Western blot analysis of TgCRT and PfCRT constructs expressed in *S*. *cerevisiae*. Each lane contains 40 μg of protein. Lane 1, yeast membranes for yeast expressing empty vector (EV); lane 2, PfCRT membranes; lane 3, TgCRT membranes; lane 4, PMA-TgCRT fusion membranes; lane 5, blank; lane 6, cytosol from TgCRT yeast; lane 7, cytosol from PMA-TgCRT yeast. The unmodified TgCRT is not expressed in *S*. *cerevisiae* (lane 3); however, the PMA-TgCRT fusion construct is expressed to similar levels relative to PfCRT, and is membrane localized. Lower molecular mass bands in lane 4 are proteolytic products, and can also be found in the cytosolic fraction (lane 7).
(TIF)

S9 Fig Alignment of TgCRT and PfCRT primary sequences and schematic of the PMA-TgCRT construct expressed in *S*. *cerevisiae*. (A) Alignment of TgCRT and PfCRT amino acid sequences reveals the 300 most N-terminal residues to be non-homologous, and that they do not encode any putative transmembraneous domains or inter-helical loop regions, whereas the remainder of TgCRT is highly homologous to PfCRT. Alignment analysis also revealed that the threonine residue at position 369 within TgCRT corresponds to the well-characterized lysine residue at position 76 within PfCRT (highlighted in red box). Identical and similar residues are highlighted in black and dark grey, respectively. (B) The 111 most N-terminal residues of *S*. *cerevisiae* plasma membrane ATPase (PMA; black) are fused in frame to the truncated TgCRT (dark grey) from which the first 300 codons have been deleted. The construct includes a C-terminal tag comprised of hexaHIS (H), biotin acceptor domain (B), and V5 epitope tag (V; “HBV” light grey). (C) Primary amino acid structure of PMA-TgCRT. Residues from PMA are shown in black, those from TgCRT are in shown dark grey, and those comprising the tag are shown in light grey.
(TIF)

S10 Fig The Δ*vp1* mutant did not display an enlarged VAC or reduced expression of TgSUB1. (A) Purified WT, Δ*vp1*, and Δ*vp1VP1* parasites were glued onto the surface of a slide for differential interference contrast (DIC) microscopy imaging. No enlarged concave structure was observed in the Δ*vp1* mutant. Bar = 5 μm. (B) The VAC sizes in WT, Δ*vp1*, and Δ*vp1VP1* were quantified based on TgCPL staining using the methods mentioned above. There were no differences in the VAC size among these strains. Three replicates were conducted for measurement. Bar = 2 μm. (C) The lysates of WT, Δ*vp1*, and Δ*vp1VP1* were prepared and probed against anti-SUB1 antibodies. The expression levels of *TgSUB1* were comparable between WT and Δ*vp1* strains. Statistical significance was calculated by unpaired two-tailed Student’s *t*-test. **, *p*<0.01; n.s., not significant.
(TIF)

S11 Fig Δ*crt* parasites were more sensitive to the treatment of chloroquine than WT parasites. WT, Δ*crt*, and Δ*crtCRT* parasites expressing luciferase were used to infect host cells in the presence of 100 μM or 0 μM chloroquine. The luciferase activity of each strain was measured at 2 and 26 h post-infection. The ratios of the luciferase activities determined at 26 hours over that at 2 h were plotted. The measurements were repeated in 4 replicates. Statistical significance was determined using unpaired two-tailed Student’s *t*-test. *, *p*<0.05; n.s., not significant.
(TIF)

S1 Text Description of generation of *TgCRT*-deficient and *TgCRT* complementation parasite strains. (DOCX)

S2 Text Raw data of mean ± variances for quantitative data plotted in the figures. (DOCX)

S1 Table The subset of genes which had a fold-change of more than 1.5-fold between WT and *Δcrt* strains as determined by differential gene expression analysis. The genes whose fold changes were >1.5 and *p*-values were <0.05 are listed.
(XLSX)

S2 Table Primers used in this study. (XLSX)

S3 Table The full gene list of differential gene expression analysis via transcriptomic sequencing between WT and *Δcrt* strains. (XLSX)
