# Supplementary figures and images for: An ortholog of Plasmodium falciparum chloroquine resistance transporter (PfCRT) plays a key role in maintaining the integrity of the endolysosomal system in Toxoplasma gondii to facilitate host invasion
Source: PLoS Pathog. 2019 Jun 6;15(6):e1007775. doi: 10.1371/journal.ppat.1007775 (PMC6553793; doi:10.1371/journal.ppat.1007775)

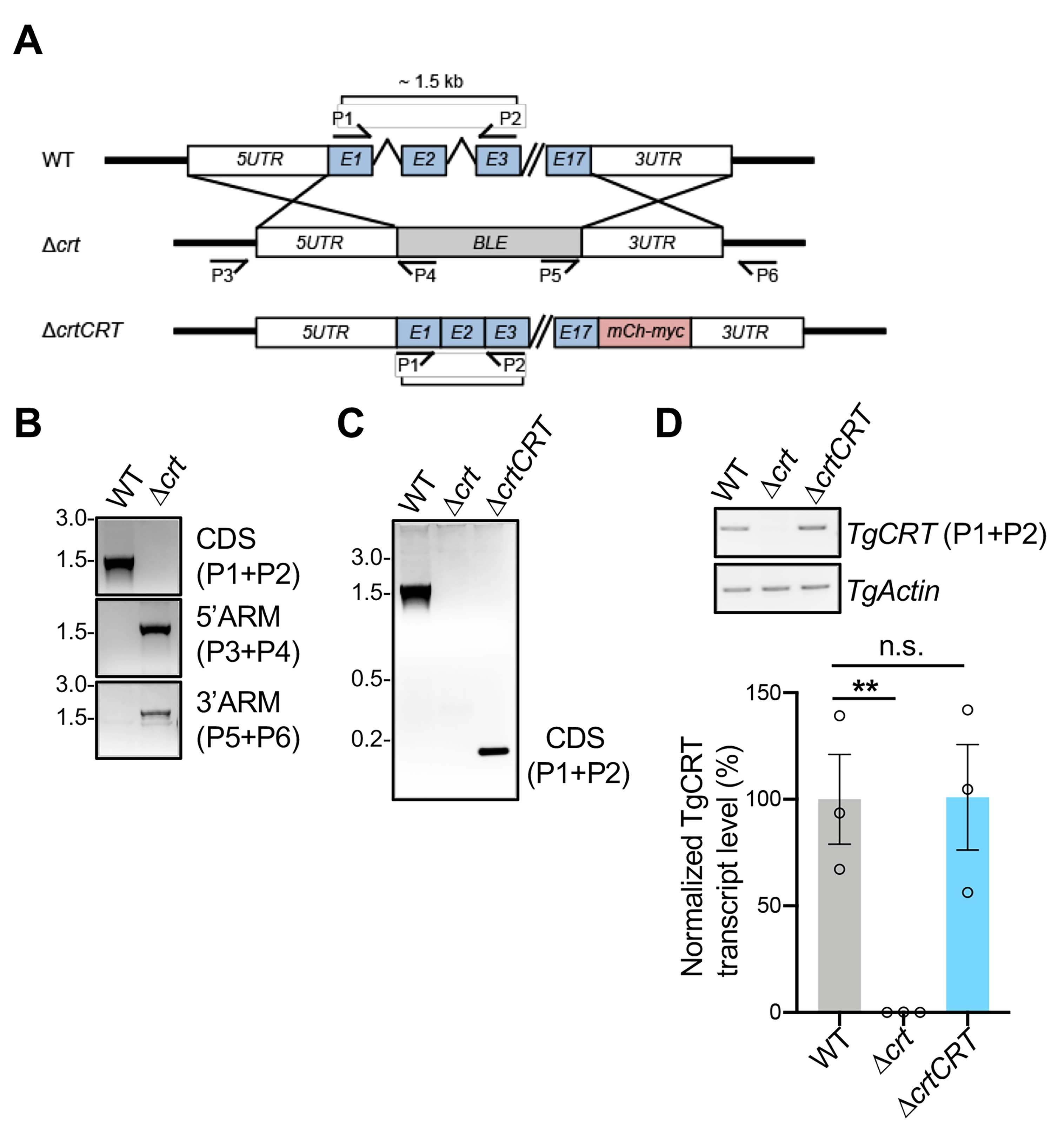

Supplement: S1 Fig — (A) Schematic illustration of the strategies for the TgCRT deletion and complementation in Toxoplasma parasites. The plasmid carrying a bleomycin resistance cassette (BLE) flanked by the TgCRT targeting sequences was transfected into WT parasites for double-crossover replacement of TgCRT to produce the Δcrt strain. The TgCRT complementation plasmid, containing the coding sequence of TgCRT fused with mCherry and 3xmyc epitope tags at its C-terminus, was introduced into the Δcrt strain to produce the ΔcrtCRT complementation strain. (B) The primers indicated in panel A were used to verify the correct replacement of TgCRT with BLE by PCR. (C) The complemented TgCRT gene was transfected into Δcrt parasites and verified by PCR. Since we complemented Δcrt with the coding sequence of TgCRT, the PCR product was a 0.2 kb fragment in the ΔcrtCRT strain, whereas showing a 1.5 kb product in the WT strain whose TgCRT gene contains the introns. (D) Transcript levels of TgCRT in the WT, Δcrt, and ΔcrtCRT strains were evaluated by quantitative PCR. Primers were designed to anneal to the exons of TgCRT and are indicated in panel A. The TgActin gene was included as a control for normalization. The quantification of transcripts was performed in three biological replicates and analyzed using unpaired two-tailed Student’s t-test. **, p<0.01; n.s., not significant. (TIF) [file ppat.1007775.s001.tif]

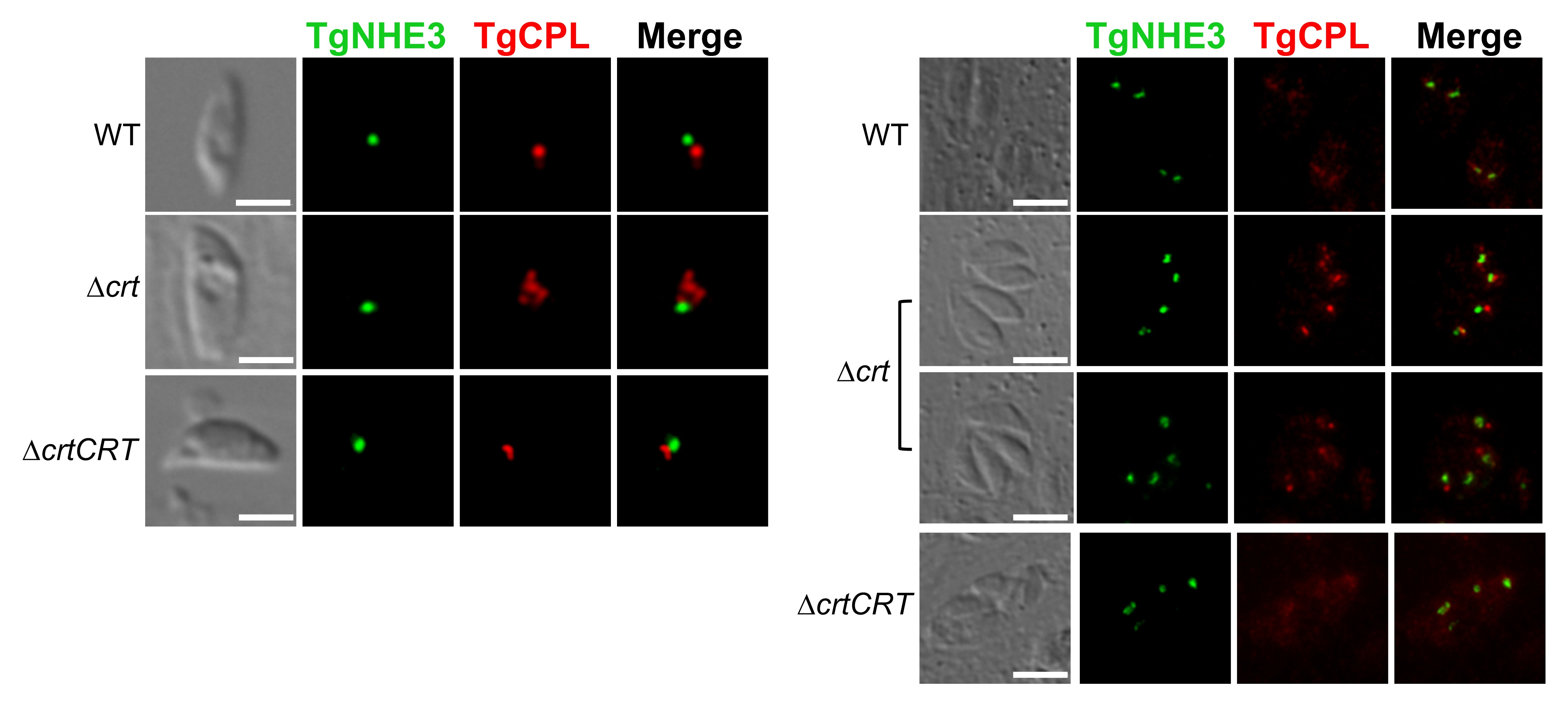

Supplement: S2 Fig — Pulse invaded and replicated parasites were co-stained with anti-TgCPL (the marker of the VAC) and anti-TgNHE3 (one marker of the ELC). The TgCRT and TgNHE3 staining in the Δcrt parasites at both stages were juxtaposed similarly to that which was seen in the WT and ΔcrtCRT strains. The scale bars in the images of pulse invaded and replicated parasites are 2 μm and 5 μm, respectively. (TIF) [file ppat.1007775.s002.tif]

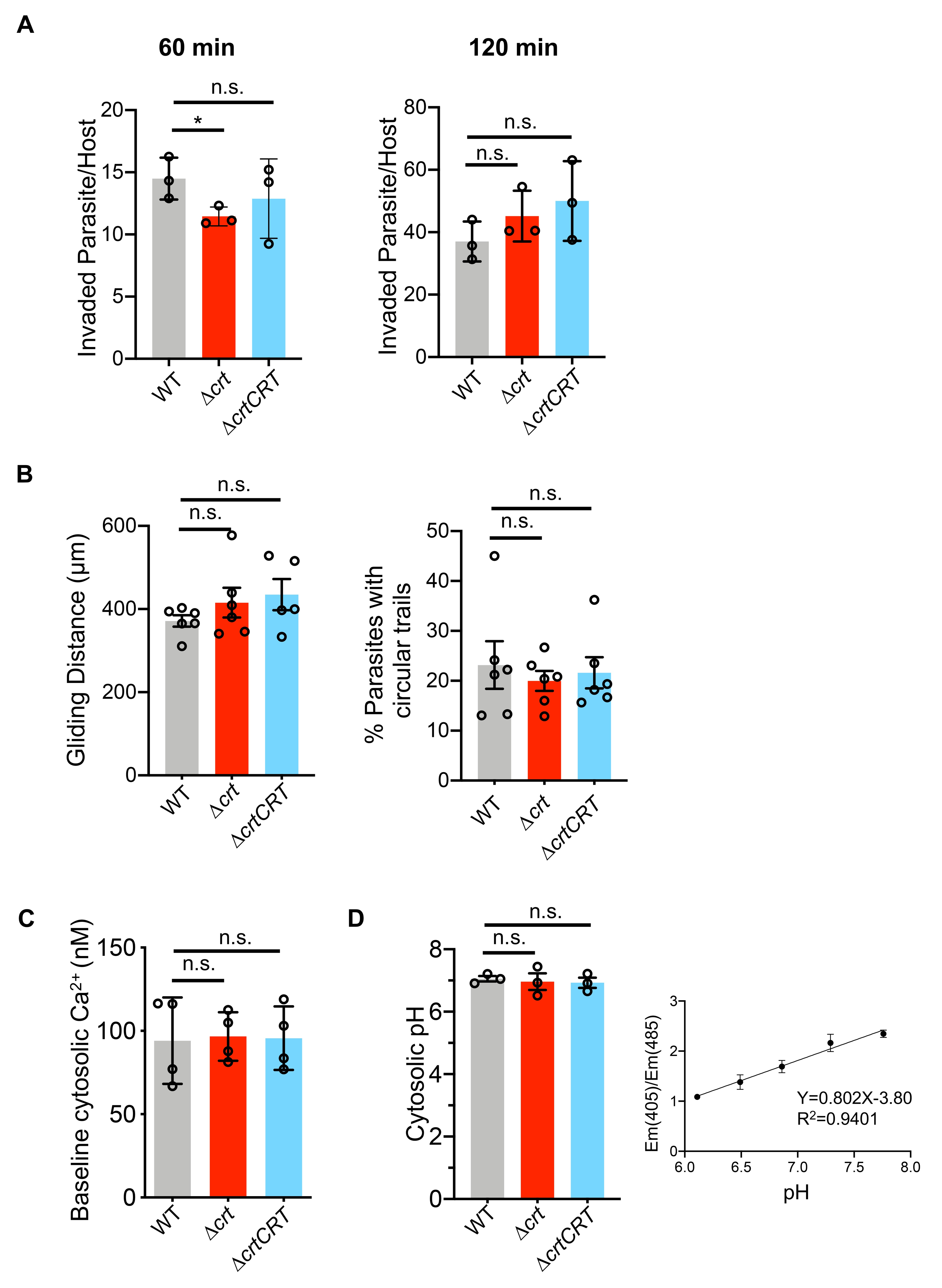

Supplement: S3 Fig — (A) The extent of the invasion defects in the Δcrt mutant was gradually minimized over time. At 60 min post-infection, there was approximately a 20% reduction in invasion in the Δcrt mutant (11.45 ± 0.76) compared to WT (14.49 ± 1.68) and ΔcrtCRT (12.88 ± 3.19) strains. At 120 min post-infection, WT, Δcrt, and ΔcrtCRT strains displayed 37.03 ± 6.42, 45.14 ± 8.14, and 50.02 ± 12.77 parasites per host cell, respectively, which did not indicate significant invasion differences among these three strains at this time point. The assay was performed in triplicate. (B) We compared the gliding distances and types of WT, Δcrt, and ΔcrtCRT strains, and did not observe significant defects in gliding motility for the Δcrt mutant. All of the assays were repeated in 5–6 replicates. (C) The baseline cytosolic calcium concentrations among these strains were evaluated by using ratiometric fluorescence measurements. Comparable calcium levels were observed in the cytoplasm of WT, Δcrt, and ΔcrtCRT parasites. Calcium quantification was repeated in 4 replicates. (D) The cytosolic pH was determined by introducing a ratiometric pH-sensitive fluorescent protein, named pHluorin 2 (PHL2) into these strains. The cytosolic pH among these strains was calculated by applying the fluorescence ratio of the PHL2 excited at 405 and 485 nm to an equation deduced from a calibration curve. Three independent replicates were performed. No cytosolic pH differences were observed among these strains. Statistical significance in all assays listed in this figure was determined using unpaired two-tailed Student’s t-test. *, p<0.05; n.s., not significant. (TIF) [file ppat.1007775.s003.tif]

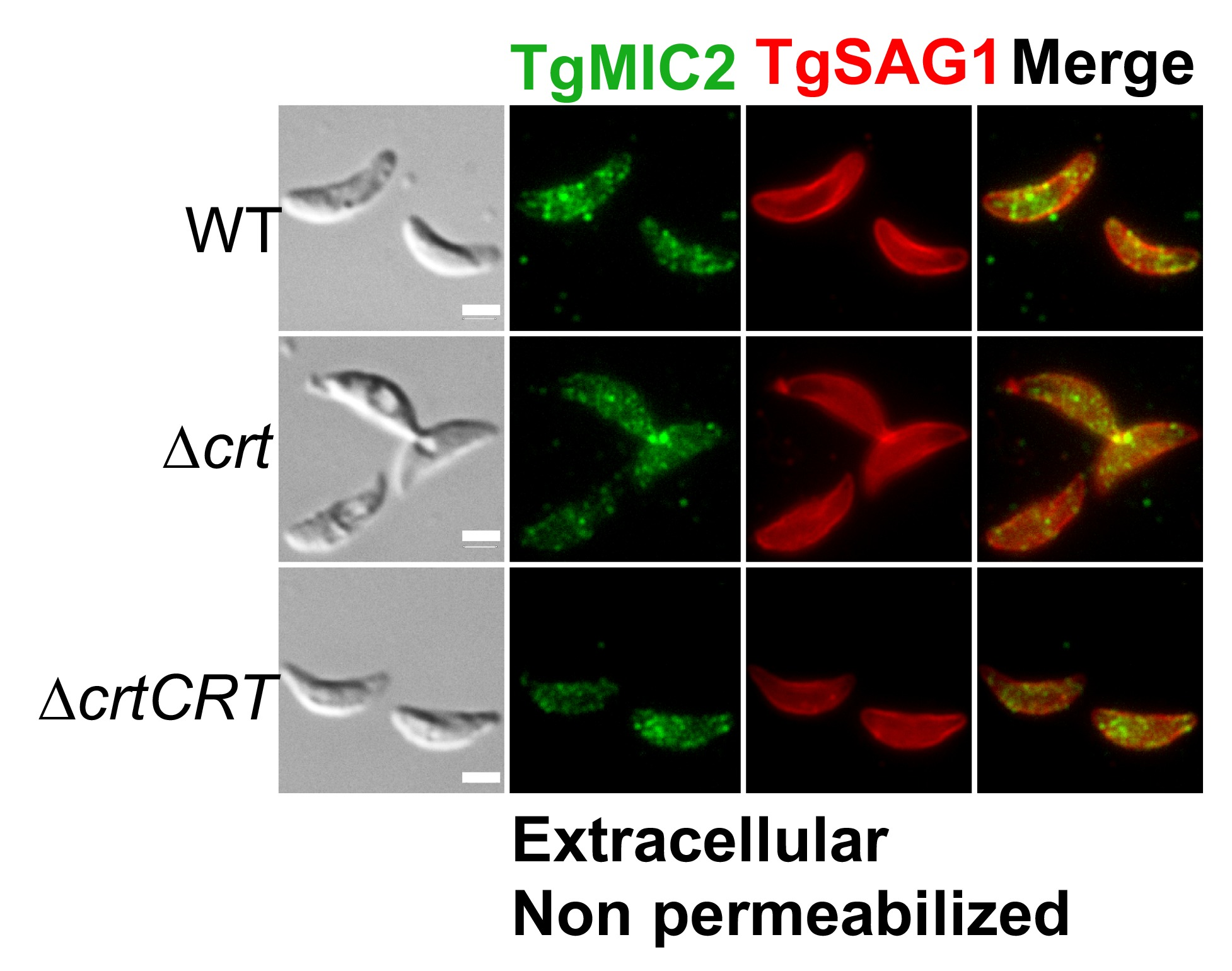

Supplement: S4 Fig — Purified, extracellular parasites that had not been permeabilized were stained with anti-TgMIC2 and anti-TgSAG1 antibodies in order to measure the retention of TgMIC2 on the parasite surface. During secretion, the TgMIC2 protein is cleaved by intramembrane rhomboid proteases, such as TgROM4. The abundance of TgMIC2 on the surface of Δcrt parasites was similar to that of the WT and ΔcrtCRT strains, indicating that there is comparable intramembrane cleavage of TgMIC2 among the parasites with or without TgCRT. (TIF) [file ppat.1007775.s004.tif]

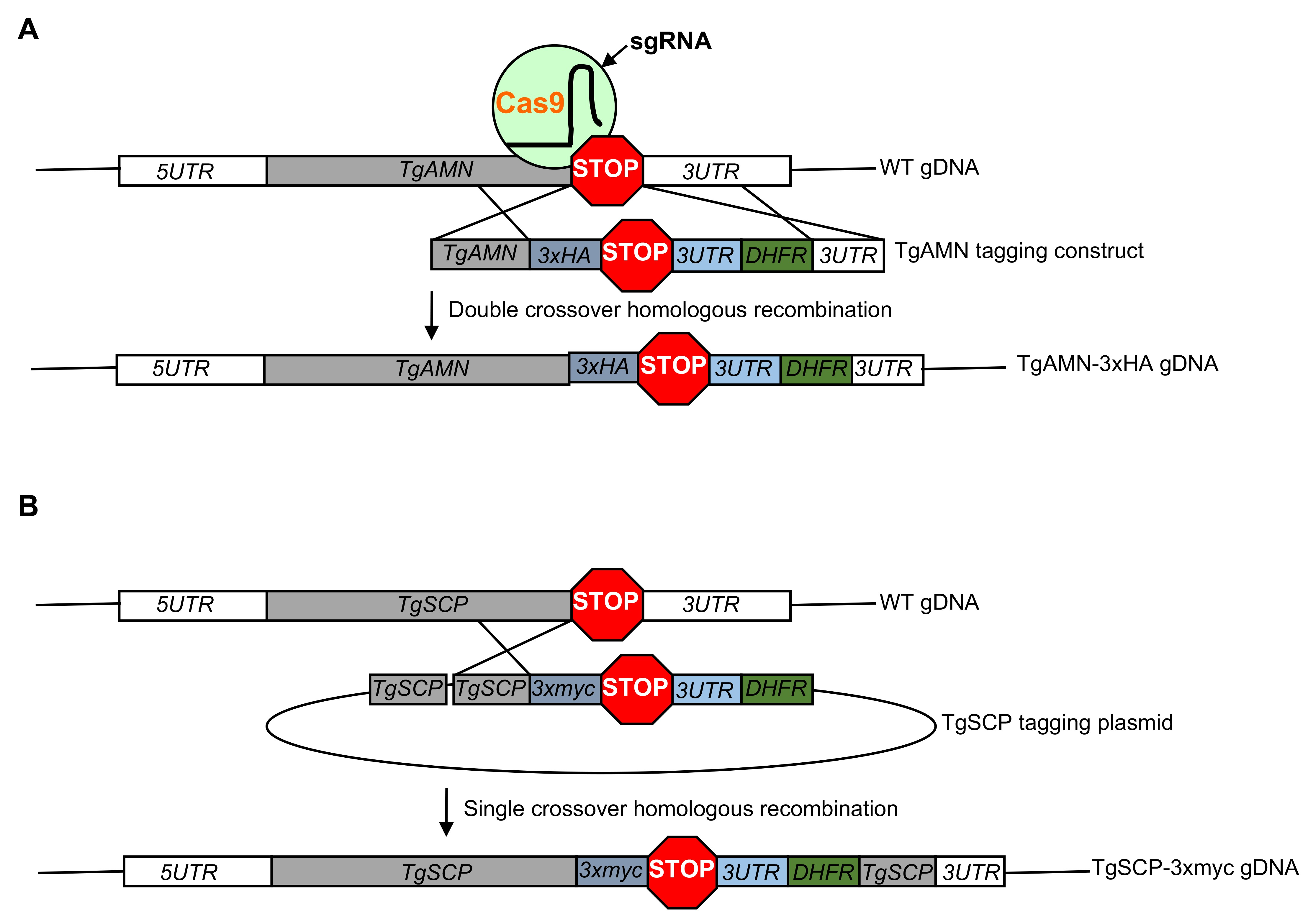

Supplement: S5 Fig — (A) The plasmids encoding Cas9 and sgRNA targeting TgAMN were co-transfected into WT parasites with the PCR product carrying a 3xHA epitope tag and a pyrimethamine resistance cassette (DHFR) flanked by 50 bp regions upstream and downstream of the stop codon of TgAMN. The 3xHA tag and the drug resistance cassette were incorporated at the C-terminus of the Toxoplasma putative aminopeptidase N via double crossover homologous recombination mediated by the CRISPR-Cas9 genome editing tool. (B) The putative Pro-Xaa serine carboxypeptidase was endogenously tagged with a 3xmyc epitope tag at its C-terminus by single crossover homologous recombination. A 1 kb region upstream of the stop codon of TgSCP was amplified and fused at the 5’-end of the 3xmyc tag to produce the TgSCP-3xmyc tagged plasmid. The 1 kb TgSCP-coding region was cleaved by an endonuclease in the middle prior to transfection to facilitate its integration. (TIF) [file ppat.1007775.s005.tif]

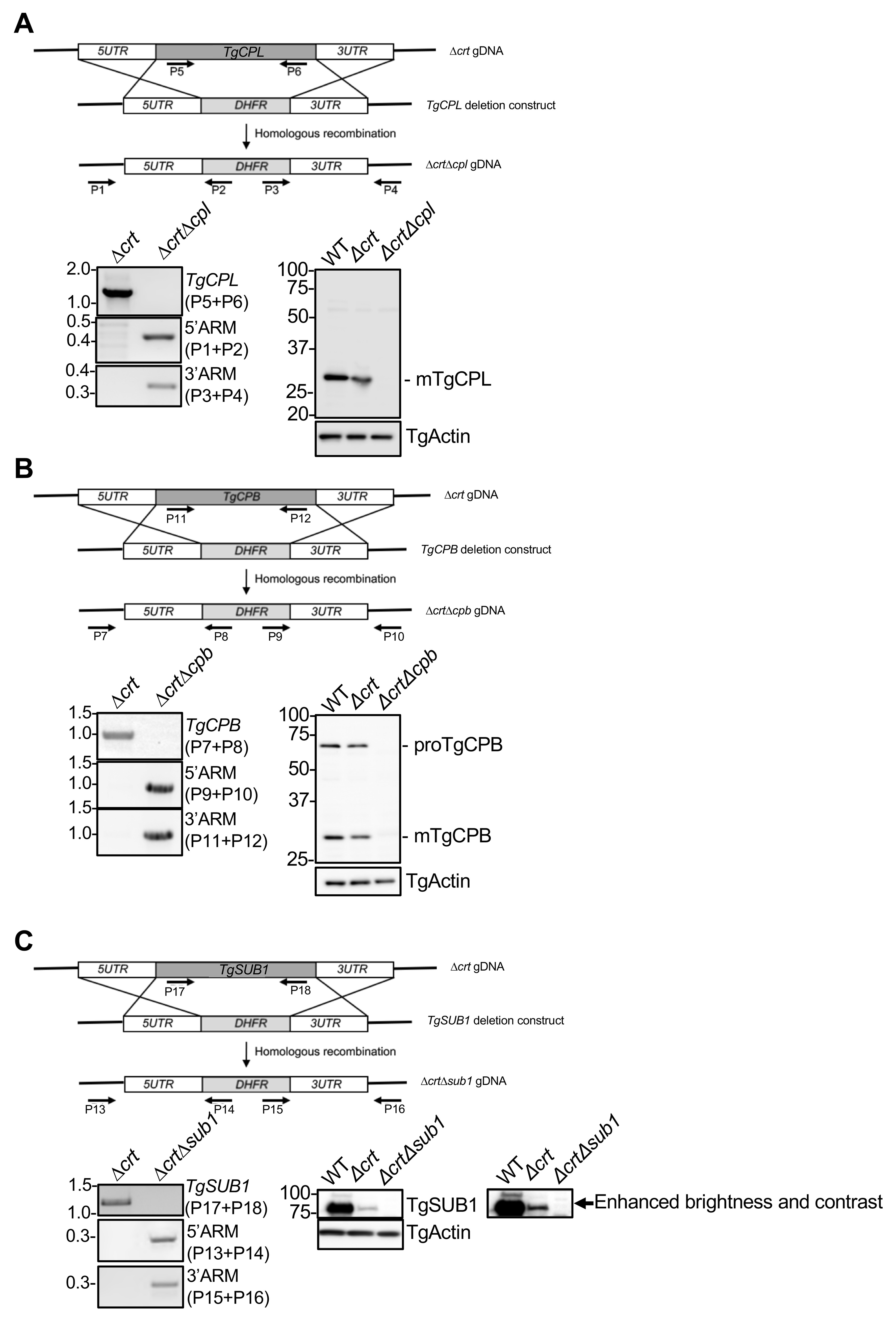

Supplement: S6 Fig — (A) Schematic illustration for the generation of the ΔcrtΔcpl mutant. A PCR product carrying a pyrimethamine resistance cassette (DHFR) flanked by 50 bps of the 5’- and 3’-untranscribed regions of TgCPL was transfected into Δcrt parasites for double-crossover replacement of TgCPL. Primers indicated in panel A were used to verify the replacement of TgCPL with DHFR via PCR and agarose gel electrophoresis. The ablation of TgCPL in ΔcrtΔcpl parasites was also confirmed by immunoblotting. (B) A similar strategy was used for the generation of the ΔcrtΔcpb mutant. The mutant was confirmed by PCR and immunoblotting. (C) The TgSUB1 gene was also genetically deleted from the Δcrt mutant by using a similar method. PCR and immunoblotting were used to confirm the deletion of TgSUB1. The TgSUB1 protein level was found to be dramatically reduced in Δcrt, but a residual amount of TgSUB1 was still observed compared to the ΔcrtΔsub1 mutant. (TIF) [file ppat.1007775.s006.tif]

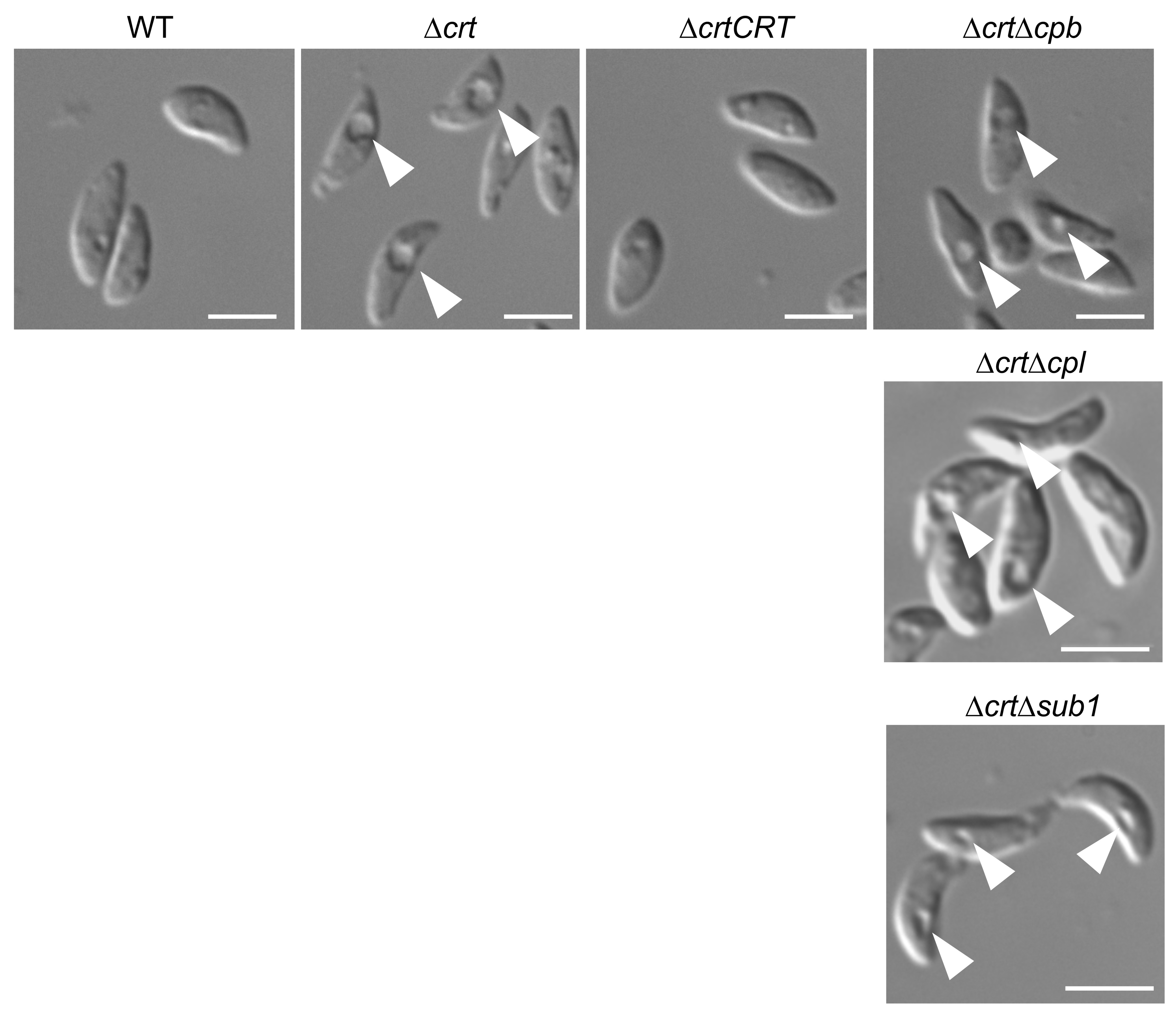

Supplement: S7 Fig — WT, Δcrt, ΔcrtCRT, ΔcrtΔcpl, ΔcrtΔcpb, and ΔcrtΔsub1 parasites were purified and attached to the surface of a slide for differential interference contrast (DIC) microscopy imaging. Although the crtΔcpl, ΔcrtΔcpb, and ΔcrtΔsub1 mutants still showed an enlarged concave subcellular structure (indicated by the arrow), their sizes were significantly smaller than those in the Δcrt mutant. Scale bar = 5 μm. (TIF) [file ppat.1007775.s007.tif]

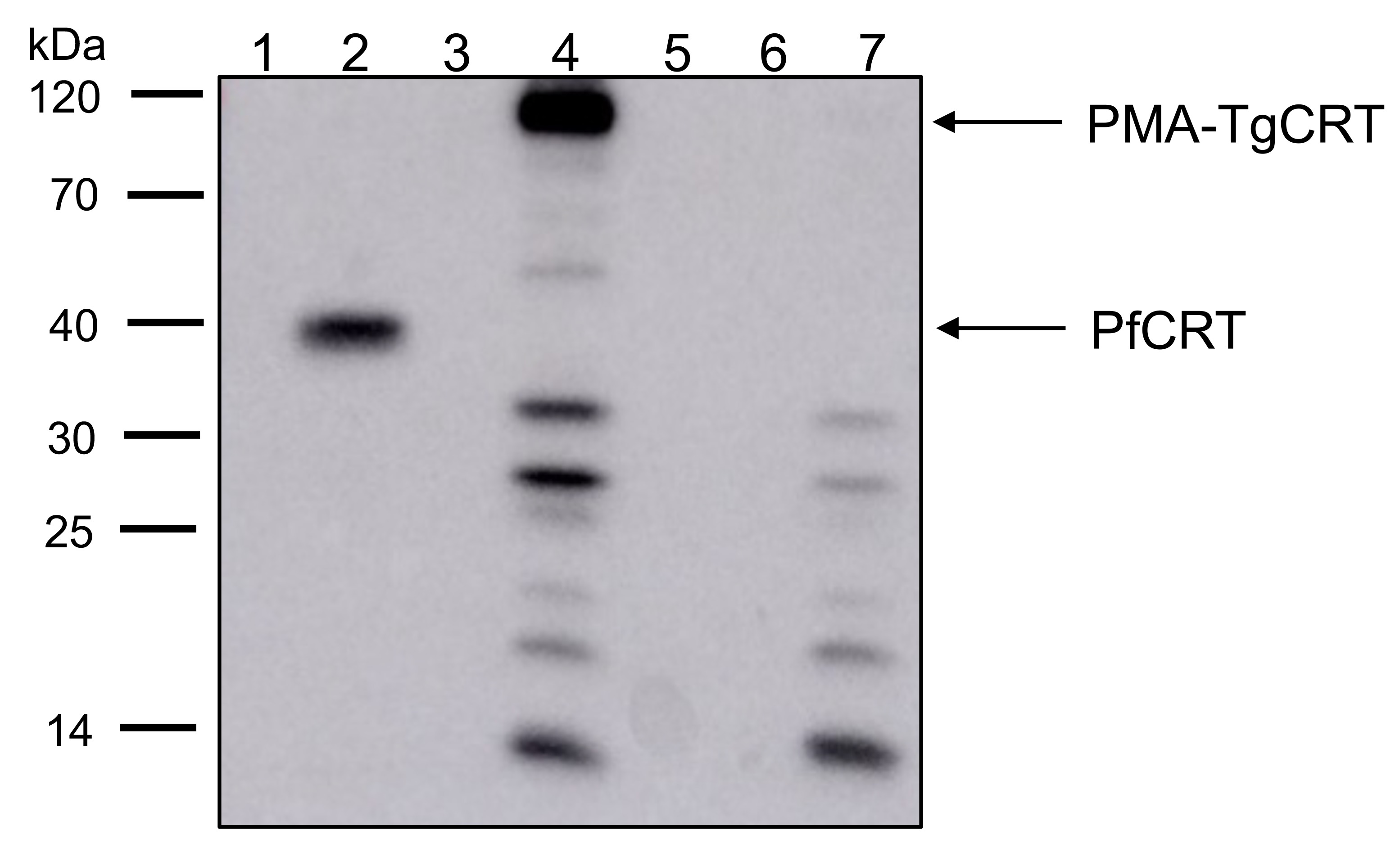

Supplement: S8 Fig — Each lane contains 40 μg of protein. Lane 1, yeast membranes for yeast expressing empty vector (EV); lane 2, PfCRT membranes; lane 3, TgCRT membranes; lane 4, PMA-TgCRT fusion membranes; lane 5, blank; lane 6, cytosol from TgCRT yeast; lane 7, cytosol from PMA-TgCRT yeast. The unmodified TgCRT is not expressed in S. cerevisiae (lane 3); however, the PMA-TgCRT fusion construct is expressed to similar levels relative to PfCRT, and is membrane localized. Lower molecular mass bands in lane 4 are proteolytic products, and can also be found in the cytosolic fraction (lane 7). (TIF) [file ppat.1007775.s008.tif]

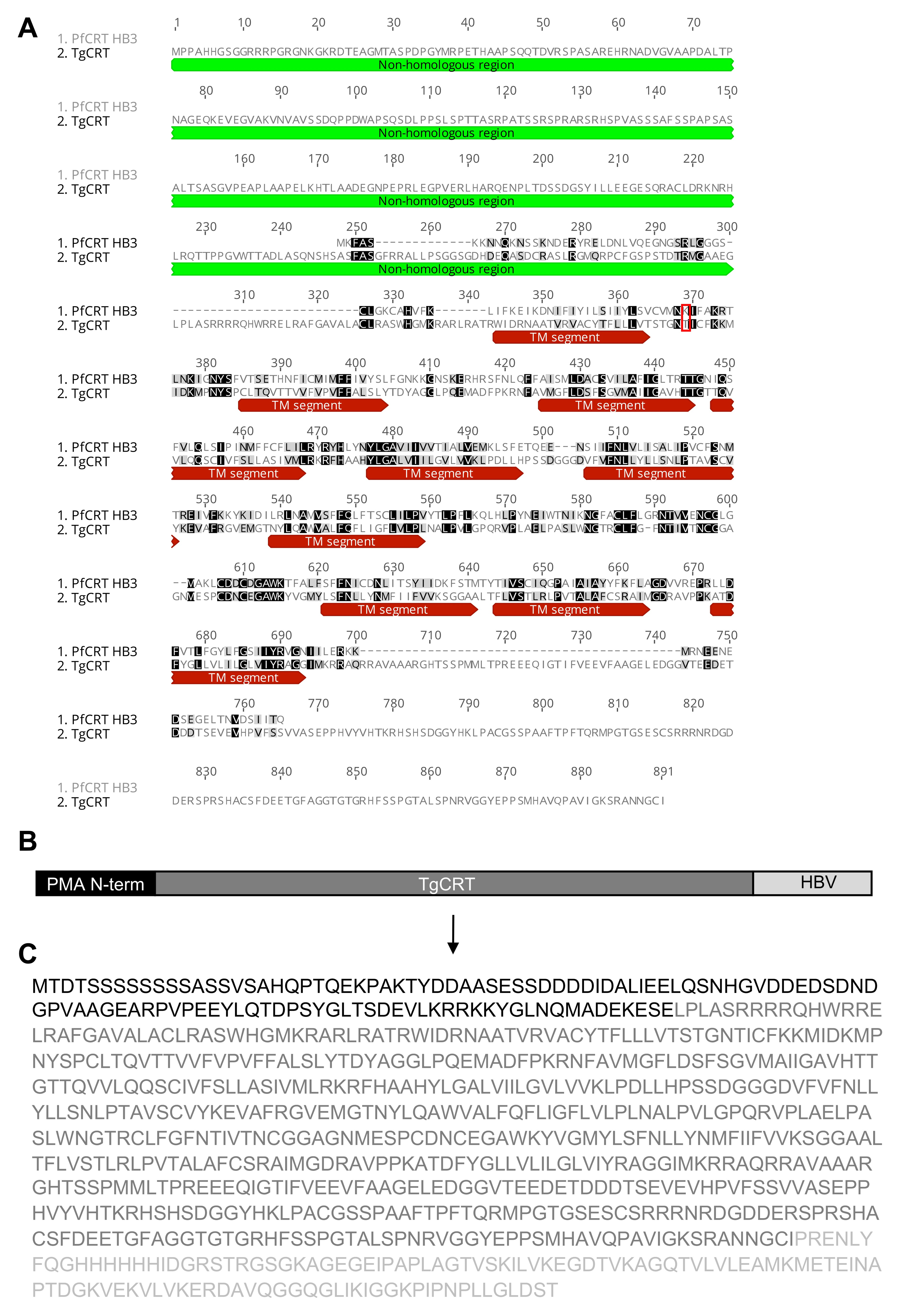

Supplement: S9 Fig — (A) Alignment of TgCRT and PfCRT amino acid sequences reveals the 300 most N-terminal residues to be non-homologous, and that they do not encode any putative transmembraneous domains or inter-helical loop regions, whereas the remainder of TgCRT is highly homologous to PfCRT. Alignment analysis also revealed that the threonine residue at position 369 within TgCRT corresponds to the well-characterized lysine residue at position 76 within PfCRT (highlighted in red box). Identical and similar residues are highlighted in black and dark grey, respectively. (B) The 111 most N-terminal residues of S. cerevisiae plasma membrane ATPase (PMA; black) are fused in frame to the truncated TgCRT (dark grey) from which the first 300 codons have been deleted. The construct includes a C-terminal tag comprised of hexaHIS (H), biotin acceptor domain (B), and V5 epitope tag (V; “HBV” light grey). (C) Primary amino acid structure of PMA-TgCRT. Residues from PMA are shown in black, those from TgCRT are in shown dark grey, and those comprising the tag are shown in light grey. (TIF) [file ppat.1007775.s009.tif]

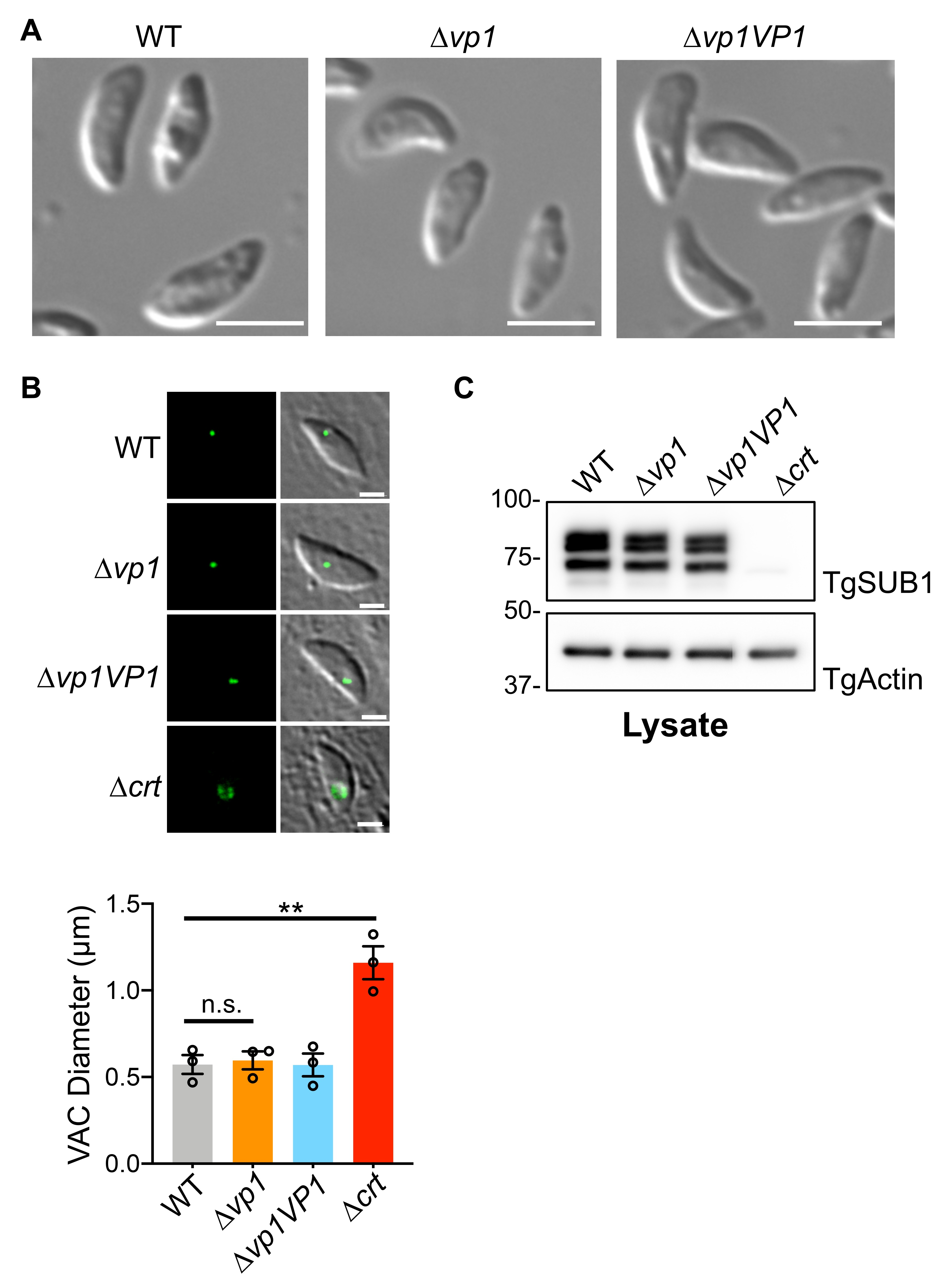

Supplement: S10 Fig — (A) Purified WT, Δvp1, and Δvp1VP1 parasites were glued onto the surface of a slide for differential interference contrast (DIC) microscopy imaging. No enlarged concave structure was observed in the Δvp1 mutant. Bar = 5 μm. (B) The VAC sizes in WT, Δvp1, and Δvp1VP1 were quantified based on TgCPL staining using the methods mentioned above. There were no differences in the VAC size among these strains. Three replicates were conducted for measurement. Bar = 2 μm. (C) The lysates of WT, Δvp1, and Δvp1VP1 were prepared and probed against anti-SUB1 antibodies. The expression levels of TgSUB1 were comparable between WT and Δvp1 strains. Statistical significance was calculated by unpaired two-tailed Student’s t-test. **, p<0.01; n.s., not significant. (TIF) [file ppat.1007775.s010.tif]

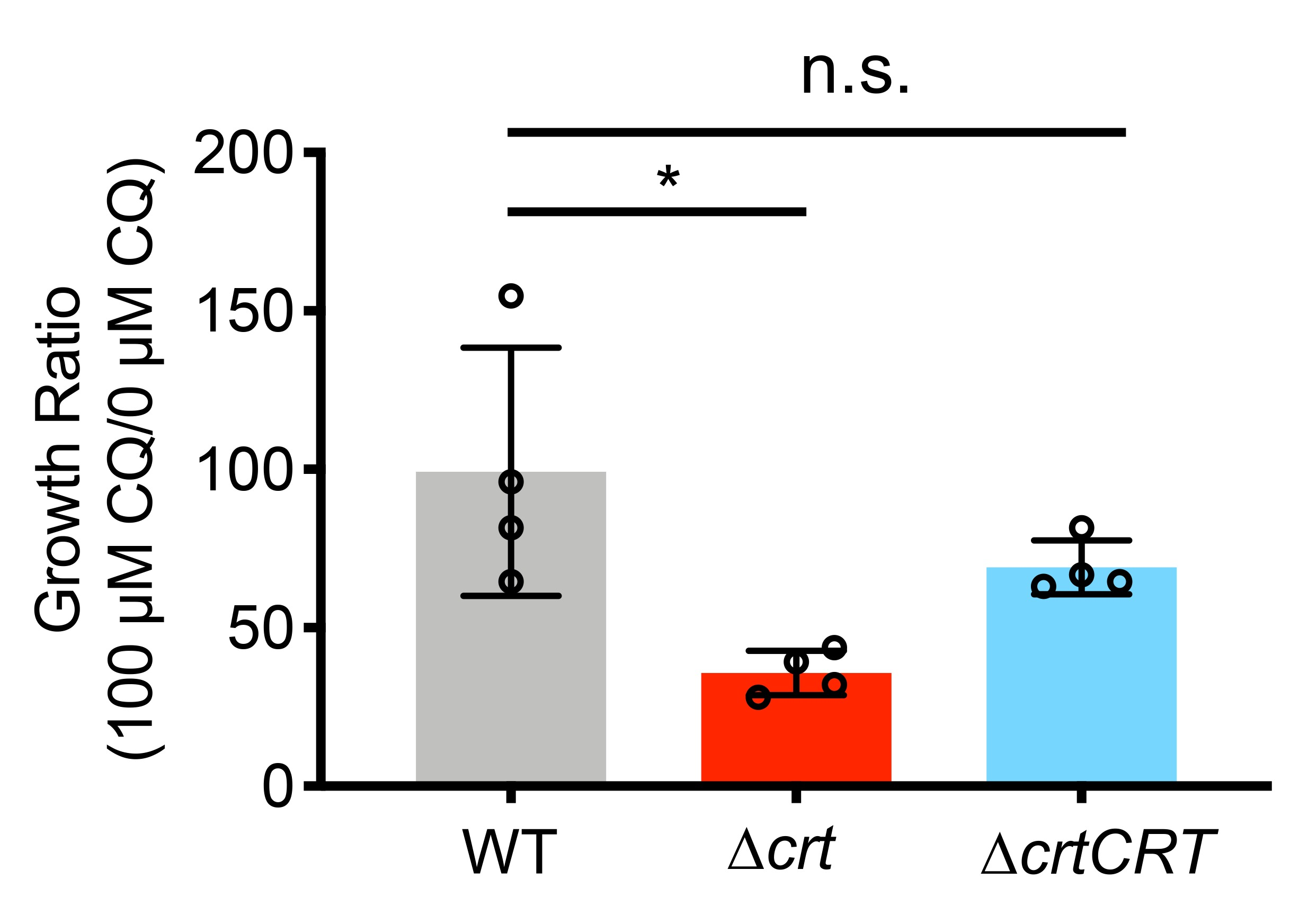

Supplement: S11 Fig — WT, Δcrt, and ΔcrtCRT parasites expressing luciferase were used to infect host cells in the presence of 100 μM or 0 μM chloroquine. The luciferase activity of each strain was measured at 2 and 26 h post-infection. The ratios of the luciferase activities determined at 26 hours over that at 2 h were plotted. The measurements were repeated in 4 replicates. Statistical significance was determined using unpaired two-tailed Student’s t-test. *, p<0.05; n.s., not significant. (TIF) [file ppat.1007775.s011.tif]
