# Supplementary material for: An ortholog of Plasmodium falciparum chloroquine resistance transporter (PfCRT) plays a key role in maintaining the integrity of the endolysosomal system in Toxoplasma gondii to facilitate host invasion
Source: PLoS Pathog. 2019 Jun 6;15(6):e1007775. doi: 10.1371/journal.ppat.1007775 (PMC6553793; doi:10.1371/journal.ppat.1007775)
Supplement: S1 Text — (DOCX) [file ppat.1007775.s012.docx]

**Supplemental Information:**

**1. Deletion of *TgCRT* in *Toxoplasma gondii*.**

We PCR amplified ~1.5-kb regions of the 5’- and 3’-untranscribed regions (UTRs) of *TgCRT*, and flanked both at both 5’- and 3’-ends with a bleomycin (*BLE*) resistance cassette to assemble a *TgCRT* knockout plasmid. The resulting construct was transfected into WT parasites to replace the entire *TgCRT* gene with a *BLE* resistance cassette by double crossover homologous recombination **(S1A Fig)**. PCR analysis was performed to test for proper integration and detected the presence of the *BLE* cassette and the loss of *TgCRT* as shown in the scheme for the generation of the *TgCRT* knockout **(S1A Fig and Table 1)**. Amplification of ~1.6 kb fragments at both the 5’- and 3’-end integration regions (5’- and 3’-ARMs) were observed in the *TgCRT* knockout (∆*crt*), and a ~1.5 kb *TgCRT* coding region was also missing within ∆*crt* **(S1B Fig)**.

To complement loss of *TgCRT*, we modified the pTub-TgCRT-mCherry-3xmyc plasmid (a kind gift from Dr. Giel van Dooren), which over-expresses a mCherry-3xmyc epitope-tagged *TgCRT* under control of the *Toxoplasma* tubulin promoter. A 1 kb DNA region containing the cognate *TgCRT* promoter was PCR-amplified and used to replace the tubulin promoter to provide similar transcription of complemented versus endogenous *TgCRT*. The same primer set used to detect loss of *TgCRT* in the ∆*crt* strain in Fig 1B was used to confirm integration of exogenously introduced *TgCRT*. Since the complemented *TgCRT* gene lacks introns, a ~0.2 kb PCR product was observed in the ∆*crtCRT* complementation strain, whereas a ~1.5 kb fragment was found for the WT strain **(S1C Fig)**. To confirm that transcription of *TgCRT* in the ∆*crtCRT* strain was comparable to endogenous levels, SYBR^®^ Green-based quantitative PCR (qPCR) was used to quantify messenger *TgCRT* RNA in WT, ∆*crt*, and ∆*crtCRT* strains. No *TgCRT* transcripts were observed in ∆*crt*, further validating successful gene disruption. *TgCRT* transcript levels were similar between the WT and ∆*crtCRT* strains **(S1D Fig)**. These data showed successful ablation of the *TgCRT* gene in *Toxoplasma* parasites, and properly restored expression with exogenously introduced *TgCRT* for the ∆*crtCRT* strain.
