# Supplementary material for: An ortholog of Plasmodium falciparum chloroquine resistance transporter (PfCRT) plays a key role in maintaining the integrity of the endolysosomal system in Toxoplasma gondii to facilitate host invasion
Source: PLoS Pathog. 2019 Jun 6;15(6):e1007775. doi: 10.1371/journal.ppat.1007775 (PMC6553793; doi:10.1371/journal.ppat.1007775)
Supplement: S2 Text — (DOCX) [file ppat.1007775.s013.docx]

**Figure 1.**

Panel B. Measurement of the VAC size in the parasites. Please refer to the mean ± SEM listed in the figure.

Panel C. Measurement of the Pearson’s correlation coefficient (PCC) for the VAC and ELC. Data are presented as mean ± SD.

TgCPL vs. proTgM2AP

Pulse invasion

WT: 0.336 ± 0.149

∆*crt*: 0.643 ± 0.128

∆*crtCRT*: 0.400 ± 0.208

Replicated

WT: 0.049 ± 0.373

∆*crt*: 0.499 ± 0.326

∆*crtCRT*: 0.077 ± 0.385

TgCPL vs. TgVP1

Pulse invasion

WT: 0.522 ± 0.185

∆*crt*: 0.736 ± 0.089

∆*crtCRT*: 0.582 ± 0.136

Replicated

WT: 0.298 ± 0.343

∆*crt*: 0.555 ± 0.223

∆*crtCRT*: 0.125 ± 0.352

**Figure 2.**

Panel A. Parasite invasion at 30 min post-infection. Please refer to the mean ± SD listed in the figure caption.

Panel B. Parasite attachment assay. Data are presented as mean ± SD.

WT: 1.38 ± 0.19 parasites per host cell

∆*crt*: 0.79 ± 0.09 parasites per host cell

∆*crtCRT*: 1.71 ± 0.14 parasites per host cell

Panel C. Parasite replication assay. Please refer to the mean ± SD values of the parasites per parasitophorous vacuole at 28 and 40 hrs post-infection listed in the figure.

For the luciferase based assay, data are presented as mean ± SD. The growth data at 24, 48, and 72 hrs post-infection were normalized against that at 2 hrs and are presented as fold-change.

24 hrs

WT: 15.6 ± 2.6

∆*crt*: 13.7 ± 2.7

∆*crtCRT*: 13.2 ± 1.0

48 hrs

WT: 200.2 ± 23.9

∆*crt*: 159.2 ± 16.5

∆*crtCRT*: 171.2 ± 10.4

72 hrs

WT: 1078.0 ± 163.2

∆*crt*: 1176.0 ± 304.2

∆*crtCRT*: 1259.0 ± 57.8

Panel D. Parasite egress assay. Data are normalized against host cells that were fully lysed using Triton X-100. The data are presented as mean ± SEM.

WT: 32.2 ± 3.3%

∆*crt*: 34.4 ± 7.0%

∆*crtCRT*: 35.6 ± 5.8%

**Figure 3.**

Panel A. Quantification of protein levels for micronemal proteins in the parasite lysate. Expression of micronemal proteins was normalized against the TgActin level. The data are presented as mean ± SD.

TgMIC2/TgActin

WT: 3.46 ± 0.25

∆*crt*: 3.81 ± 0.52

∆*crtCRT*: 3.29 ± 1.17

TgM2AP/TgActin

WT: 2.34 ± 0.44

∆*crt*: 3.14 ± 1.21

∆*crtCRT*: 2.51 ± 0.49

TgMIC5/TgActin

WT: 2.64 ± 0.30

∆*crt*: 3.52 ± 0.90

∆*crtCRT*: 2.66 ± 0.51

Panel B. Quantification of the secretion of micronemal proteins in the parasite ESAs. Abundance of micronemal proteins was normalized against the TgGRA7 level. The data are presented as mean ± SD.

**Constitutive ESA**

TgMIC2/TgGRA7

WT: 0.36 ± 0.03

∆*crt*: 0.21 ± 0.09

∆*crtCRT*: 0.31 ± 0.06

TgM2AP/TgGRA7

WT: 3.61 ± 0.27

∆*crt*: 2.66 ± 0.57

∆*crtCRT*: 2.97 ± 0.92

TgMIC5/TgGRA7

WT: 0.69 ± 0.11

∆*crt*: 0.57 ± 0.07

∆*crtCRT*: 0.58 ± 0.15

**Induced ESA**

TgMIC2/TgGRA7

WT: 3.41 ± 0.46

∆*crt*: 0.64 ± 0.65

∆*crtCRT*: 2.57 ± 2.12

TgM2AP/TgGRA7

WT: 2.51 ± 0.59

∆*crt*: 1.31 ± 0.11

∆*crtCRT*: 3.18 ± 1.18

TgMIC5/TgGRA7

WT: 0.72 ± 0.16

∆*crt*: 0.44 ± 0.06

∆*crtCRT*: 0.57 ± 0.11

**Figure 4.**

Panel D. Quantification of protein levels for TgSUB1 in the parasite lysate. Expression of TgSUB1 was normalized against the TgActin level. The data are presented as mean ± SD.

TgSUB1/TgActin

WT: 9.25 ± 1.03

∆*crt*: 1.29 ± 0.35

∆*crtCRT*: 12.36 ± 3.24

Panel E. qPCR analysis of the *TgSUB1* transcript level. Data are presented as mean ± SEM.

WT: 100.0% ± 30.4%

∆*crt*: 7.8% ± 2.4%

∆*crtCRT*: 153.5% ± 30.5%

**Figure 5**

Panel B. qPCR analysis of the transcript levels for endolysosomal proteases. Data are presented as mean ± SEM.

*TgAMN*

WT: 100.0% ± 10.8%

∆*crt*: 49.5% ± 5.3%

∆*crtCRT*: 180.6% ± 44.8%

*TgSCP*

WT: 100.0% ± 2.7%

∆*crt*: 79.4% ± 3.8%

∆*crtCRT*: 87.1% ± 11.4%

*TgASP1*

WT: 100.0% ± 13.7%

∆*crt*: 52.8% ± 4.1%

∆*crtCRT*: 121.2% ± 30.9%

*TgICEL*

WT: 100.0% ± 1.5%

∆*crt*: 108.9% ± 14.5%

∆*crtCRT*: 122.6% ± 6.8%

*TgCPL*

WT: 100.0% ± 6.7%

∆*crt*: 106.0% ± 14.8%

∆*crtCRT*: 113.8% ± 21.3%

*TgCPB*

WT: 100.0% ± 4.5%

∆*crt*: 86.3% ± 1.0%

∆*crtCRT*: 128.6% ± 19.7%

Panel C. Quantification of the protein levels for TgCPL and TgCPB in the parasite lysate. Expression of TgCPL and TgCPB was normalized against the TgActin level. The data are presented as mean ± SD.

TgCPL/TgActin

WT: 0.87 ± 0.10

∆*crt*: 0.51 ± 0.12

∆*crtCRT*: 0.85 ± 0.26

TgCPB/TgActin

WT: 1.84 ± 0.28

∆*crt*: 0.74 ± 0.24

∆*crtCRT*: 1.99 ± 0.58

**Figure 6.**

Panel B. Measurement of the VAC sizes (TgCPB and TgCPL staining) in the ∆*crt*∆*cpl*, ∆*crt*∆*cpb*, and ∆*crt*∆*sub1* parasites. Please refer to the mean ± SEM listed in the figure.

Panel C. Percentage of parasites showing co-localization of the VAC and ELC. Data are presented as mean ± SD.

WT: 19.1% ± 5.4%

∆*crt*: 62.5% ± 4.1%

∆*crtCRT*: 25.1% ± 7.6%

∆*crt∆cpb*: 44.2% ± 6.3%

Panel F. Invasion efficiency evaluation of ∆*crt*∆*cpb* at 30 min post-infection. Please refer to the mean ± SD listed in the figure caption.

**Figure 7.**

Panel B. Measurement of the VAC sizes in the WT, ∆*crt*, ∆crtCRT, and ∆*crtCRT^T369K^* parasites. Please refer to the mean ± SEM listed in the figure.

**S1. Figure**.

Panel D. qPCR analysis of the *TgCRT* transcript level. Data are presented as mean ± SEM.

WT: 100.0% ± 21.0%

∆*crt*: 0.11% ± 0.01%

∆*crtCRT*: 101.0% ± 24.8%

**S3. Figure.**

Panel A. Parasite invasion at 60 and 120 min post -infection. Please refer to the mean ± SD listed in the figure caption.

Panel B. Motility measurement. Data are presented as mean ± SEM.

WT: 371.5 ± 13.7 µm

∆*crt*: 415.3 ± 35.8 µm

∆*crtCRT*: 434.7 ± 37.6 µm

Percentage of the circular migration form. Data are presented as mean ± SEM.

WT: 23.2 ± 4.8%

∆*crt*: 20.0 ± 2.0 %

∆*crtCRT*: 21.6 ± 3.1%

Panel C. Baseline cytosolic calcium concentration determination in the parasites. Data are presented as mean ± SEM.

WT: 94.1 ± 13.0 nM

∆*crt*: 96.7 ± 7.3 nM

∆*crtCRT*: 95.6 ± 9.5 nM

Panel D. Baseline cytosolic pH determination in the parasites. Data are presented as mean ± SEM.

WT: 7.06 ± 0.09

∆*crt*: 6.96 ± 0.27

∆*crtCRT*: 6.93 ± 0.16

**S10. Figure.**

Panel B. Quantification of the VAC size in the ∆*vp1* mutant. Data are presented as mean ± SEM.

WT: 0.57 ± 0.05 µm

∆*vp1*: 0.60 ± 0.05 µm

∆*vp1VP1*: 0.57 ± 0.07 µm

∆*crt*: 1.16 ± 0.09 µm

**S11. Figure.**

Relative growth rates of parasites grown in medium containing 100 µM chloroquine compared to parasites grown in medium without chloroquine at 26 hrs post-infection. Data are presented as mean ± SD.

WT: 99.2 ± 19.6%

∆*crt*: 35.8 ± 3.5%

∆*crtCRT*: 69.0 ± 4.3%
